# Comparative genomic analysis of the *Tribolium *immune system

**DOI:** 10.1186/gb-2007-8-8-r177

**Published:** 2007-08-29

**Authors:** Zhen Zou, Jay D Evans, Zhiqiang Lu, Picheng Zhao, Michael Williams, Niranji Sumathipala, Charles Hetru, Dan Hultmark, Haobo Jiang

**Affiliations:** 1 Department of Entomology and Plant Pathology, Oklahoma State University, Stillwater, OK 74078, USA; 2 USDA-ARS Bee Research Laboratory, Beltsville, MD 20705, USA; 3 Umeå Centre for Molecular Pathogenesis, Umeå University, Umeå S-901 87, Sweden; 4 Institut Biol Moléc Cell, CNRS, Strasbourg 67084, France

## Abstract

**Background:**

*Tribolium castaneum *is a species of Coleoptera, the largest and most diverse order of all eukaryotes. Components of the innate immune system are hardly known in this insect, which is in a key phylogenetic position to inform us about genetic innovations accompanying the evolution of holometabolous insects. We have annotated immunity-related genes and compared them with homologous molecules from other species.

**Results:**

Around 300 candidate defense proteins are identified based on sequence similarity to homologs known to participate in immune responses. In most cases, paralog counts are lower than those of *Drosophila melanogaster *or *Anopheles gambiae *but are substantially higher than those of *Apis mellifera*. The genome contains probable orthologs for nearly all members of the Toll, IMD, and JAK/STAT pathways. While total numbers of the clip-domain serine proteinases are approximately equal in the fly (29), mosquito (32) and beetle (30), lineage-specific expansion of the family is discovered in all three species. Sixteen of the thirty-one serpin genes form a large cluster in a 50 kb region that resulted from extensive gene duplications. Among the nine Toll-like proteins, four are orthologous to *Drosophila *Toll. The presence of scavenger receptors and other related proteins indicates a role of cellular responses in the entire system. The structures of some antimicrobial peptides drastically differ from those in other orders of insects.

**Conclusion:**

A framework of information on *Tribolium *immunity is established, which may serve as a stepping stone for future genetic analyses of defense responses in a nondrosophiline genetic model insect.

## Abstract 2

The annotation, and comparison with homologous genes in other species, of immunity-related genes in the Tribolium castaneum genome allowed the identification of around 300 candidate defense proteins, and revealed a framework of information on Tribolium immunity.

## Background

*Tribolium *beetles harbor a range of natural pathogens and parasites, from bacteria to fungi, microsporidians and tapeworms [1,2]. There is good evidence for genetic variation in resistance to the tapeworm and a linked cost of resistance in terms of growth and reproduction [3]. Cross-generational transfer of immune traits [4] may occur in *Tenebrio molitor*, a close relative of *Tribolium castaneum*. RNA interference experiments demonstrate that *Tribolium *laccase-2 is responsible for cuticle pigmentation and sclerotization [5]. While these observations are interesting, our knowledge of the genetic constituents of *Tribolium *immunity is almost blank at the cellular and molecular levels, in contrast to the vast amount of information regarding *Drosophila melanogaster *and *Anopheles gambiae *defense responses [6,7]. Given the high efficiency of RNA interference and powerful tools of molecular genetics [8], it is particularly appealing to use *T. castaneum *for the dissection of insect immune pathways. Acquired knowledge may be useful in controlling beetle pests that feed on crop plants or stored products.

In the broader field of beetle immunity, research has been focused mainly on two effector mechanisms, namely antimicrobial peptide synthesis and prophenoloxidase (proPO) activation [9]. Defensins, coleoptericins, cecropin and antifungal peptides have been isolated from coleopteran insects and characterized biochemically [10-12]. A homolog of human NF-κB (*Allomyrina dichotoma *Rel A) up-regulates the transcription of a coleoptericin gene [13]. Active phenoloxidase generates quinones for melanin formation, wound healing, and microbe killing. ProPO activation has been investigated in *Holotrichia diomphalia *[14-16]. ProPO activating factor 1 (*Hd*-PPAF1) cleaves proPO to generate active phenoloxidase in the presence of *Hd*-PPAF2, the precursor of which is activated by *Hd*-PPAF3 via limited proteolysis. While all these PPAFs contain an amino-terminal clip domain, PPAF2 (in contrast to PPAF1 or PPAF3) does not have catalytic activity since its carboxy-terminal serine proteinase-like domain lacks the active site serine. A 43 kDa inhibitor down-regulates the melanization response in *H. diomphalia *[17].

To date, components of the innate immune system are hardly known in *T. castaneum *and neither is it clear how they differ from homologous molecules in the honeybee, mosquito or fruitfly [6,7,18]. This lack of knowledge does not seem to reconcile with the critical phylogenetic position of this coleopteran species, which should inform us a lot about genetic variations in the evolution of holometabolous insects. Information regarding defense responses in *T. castaneum*, a member of the largest and most diverse order of eukaryotes, is highly desirable for the biological control of crop pests and disease vectors. Consequently, we have used its newly available genome assembly to annotate immunity-related genes and analyze their phylogenetic relationships with homologous sequences from other insects. In this comparative overview of the *Tribolium *defense system, we describe plausible immune pathway models and present information regarding the molecular evolution of innate immunity in holometabolous species.

## Results and discussion

### Overview of the *Tribolium *immune system

*T. castaneum *has a sizable repertoire of immune proteins predicted to participate in various humoral and cellular responses against wounding or infection (Additional data file 1). Like other insects [6,7,19], cuticle and epithelia lining its body surfaces, tracheae and alimentary tract may serve as a physiochemical barrier and local molecular defense by producing antimicrobial peptides and reactive oxygen/nitrogen species (ROS/RNS). While this line of defense may block most pathogens, others enter the hemocoel where a coordinated acute-phase reaction could occur to immobilize and kill the opportunists. This reaction, including phagocytosis, encapsulation, coagulation and melanization, is probably mediated by hemocytes and molecules constitutively present in the circulation. These first responders may not only control minor infections but also call fat body and hematopoietic tissues for secondary responses if necessary. At the molecular level, the following events should take place in all insects, including the beetle: recognition of invading organisms by plasma proteins or cell surface receptors, extra- and intracellular signal transduction and modulation, transcriptional regulation of immunity-related genes, as well as controlled release of defense molecules.

### Pathogen recognition

**Figure 1:**
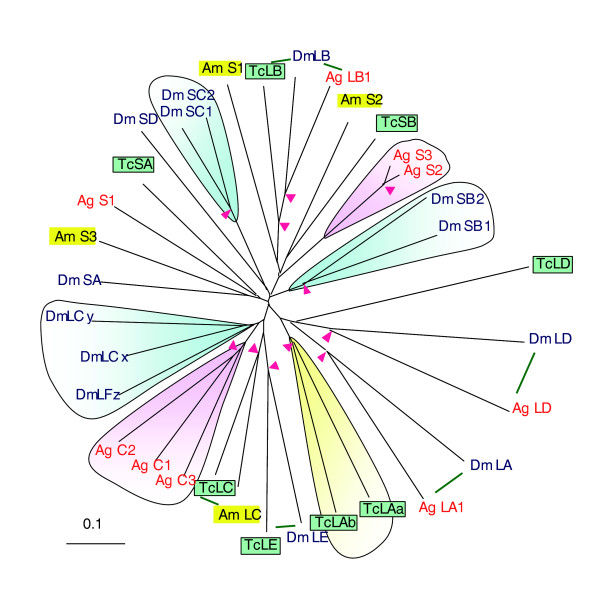
Peptidoglycan recognition proteins. The amino acid sequences from eight *Tribolium *(Tc), thirteen *Drosophila *(Dm), nine *Anopheles *(Ag), and four *Apis *(Am) PGRPs are examined. The phylogenetic tree shows family expansion in *Tribolium *(shaded yellow), *Anopheles *(shaded pink) and *Drosophila *(shaded blue). *Tc*PGRP-LA, -LC and -LD contain a transmembrane domain whereas *Tc*PGRP-SA and -SB have a signal peptide for secretion. Pink arrowheads at nodes denote bootstrap values greater than 800 from 1,000 trials. The putative 1:1 or 1:1:1 orthologs are connected by green lines. *Tc*PGRP-LB and -SB contain the key residues for an amidase activity.

Peptidoglycan recognition proteins (PGRPs) serve as an important surveillance mechanism for microbial infection by binding to Lys- and diaminopimelate-type peptidoglycans of walled bacteria [20]. Some *Drosophila *PGRPs (for example, LC and SA) are responsible for cell-mediated or plasma-based pathogen recognition; others (that is, LB and SB) may hydrolyze peptidoglycans to turn on/off immune responses [21,22]. In *T. castaneum*, PGRP-LA, -LC and -LD contain a transmembrane segment; PGRP-SA and -SB are probably secreted; PGRP-LE (without a signal peptide or transmembrane region) may exist in cytoplasm or enter the plasma via a nonclassical secretory pathway. Bootstrap analysis and domain organization clearly indicate that *Tribolium *and *Drosophila *PGRP-LEs are orthologs - so far no PGRP-LE has been identified in *Anopheles*, *Bombyx *or *Apis*. Other orthologous relationships (for example, *Tc*PGRP-LC and *Am*PGRP-LC) are also supported by the phylogenetic analysis (Figure 1). The beetle and mosquito PGRP-LA genes encode two alternative splice forms (PGRP-LAa and -LAb). Like *Drosophila *and *Anopheles*, *Tribolium *PGRP-LA and -LC genes are next to each other in the same cluster. Most of the beetle PGRPs resulted from ancient family diversification that occurred before the emergence of holometabolous insects. In contrast, gene duplication occurred several times in the lineages of mosquito and fly (Figure 1).

Multiple sequence alignment suggests that β-1,3-glucan-recognition proteins (β GRPs) and Gram-negative binding proteins (GNBPs) are descendents of invertebrate β-1,3-glucanases [23]. Lacking one or more of the catalytic residues, these homologous molecules do not possess any hydrolytic activity. They are widespread in arthropods and act in part to recognize microbial cell wall components such as β-1,3-glucan, lipoteichoic acid or lipopolysaccharide. We have identified three β GRPs in *T. castaneum*. *Tc*-β GRP1 and *Ag*GNBP-B1 through -B5 are closely related and represent a young lineage, whereas *Tc*-β GRP2 and *Tc*-β GRP3 belong to an ancient group that arose before the radiation of holometabolous insects (Additional data file 2). Since *Drosophila *has no β GRP-B and *Anopheles *has five, the presence of a single gene (encoding *Tc-β *GRP1) in the beetle can be useful for elucidating function of this orthologous group. In addition to the glucanase-like domain, members of the second group contain an amino-terminal extension of about 100 residues. In *Bombyx mori *β GRP, this region recognizes β-1,3-glucan also [24]. *M. sexta *β GRP2 binds to insoluble β-1,3-glucan and triggers a serine proteinase cascade for proPO activation [25].

C-type lectins (CTLs) comprise a wide variety of soluble and membrane-bound proteins that associate with carbohydrates in a Ca^2+^-dependent manner [26]. Some insect CTLs recognize microorganisms and enhance their clearance by hemocytes [19]. Gene duplication and sequence divergence, particularly in the sugar-interacting residues, lead to a broad spectrum of binding specificities for mannose, galactose and other sugar moieties. These proteins associate with microbes and hemocytes to form nodules [27] and stimulate melanization response [28]. *T. castaneum *encodes sixteen CTLs: ten (*Tc*-CTL1, 2, 4 through 10, and 13) with a single carbohydrate recognition domain and one (*Tc*-CTL3) with two. Five other proteins, tentatively named *Tc*-CTL11, 12, 14, 15 and 16, contain a CTL domain, a transmembrane region (except for *Tc*-CTL11), and other structural modules: CTL11 has three CUB and three EGF; CTL12 has six Ig and three FN3; CTL14 has one LDL_r_A, three CUB, ten Sushi, nineteen EGF, two discoidin, one laminin G and one hyalin repeat; CTL15 has one FTP, eleven Sushi and two EFh; CTL16 has one FTP and four Sushi. While lineage-specific expansion of the gene family is remarkable in *D. melanogaster *and *A. gambiae *[29], we have not found any evidence for that in *T. castaneum *(or *A. mellifera*): *Tc*-CTL1, 2, 5, 6, 8, 9, 12 through 16 have clear orthologs in the other insect species whereas *Tc*-CTL7, 10 and 11 are deeply rooted (Additional data file 3).

Galectins are β-galactoside recognition proteins with significant sequence similarity in their carbohydrate-binding sites characteristic of the family. *Drosophila *DL1 binds to *E. coli *and *Erwinia chrysanthemi *[30]. *Leishmania *uses a sandfly galectin as a receptor for specific binding to the insect midgut [31]. *Tc*-galectin1 has two carbohydrate recognition domains; *Tc*-galectin2 and 3 are orthologous to *Am*-galectin1 and 2, respectively (Additional data file 4).

All fibrinogen-related proteins (FREPs) contain a carboxy-terminal fibrinogen-like domain associated with different amino-terminal regions. In mammals, three classes of FREPs have been identified: ficolin, tenascins, and microfibril-associated proteins [32]. They take part in phagocytosis, wound repair, and cellular adhesion [33]. In invertebrates, FREPs are involved in cell-cell interaction, bacterial recognition, and antimicrobial responses [34-36]. The *Tribolium *genome contains seven FREP genes, which fall into three groups (Additional data file 5): the expansion of group I yielded four family members: *Tc*-FREP1 through 4. Sitting next to each other on chromosome 3, these beetle genes encode polypeptides most similar to angiopoietin-like proteins. During angiogenesis, the human plasma proteins interact with tyrosine kinase receptors (for example, Tie) and lead to wound repair and tissue regeneration [37]. In group II, *Tc*-FREP5 is orthologous to *Dm-*scabrous, which is required for Notch signaling during tissue differentiation [38]. Interestingly, Notch is also needed for proper differentiation of *Drosophila *hemocytes [39]. Group III includes *Tc*-FREP6, *Tc*-FREP7, *Ag*-FREP9 and *Dm*-CG9593. No major expansion has occurred in the beetle or honeybee, in sharp contrast to the situations in the fly and mosquitoes - there are 61 FREP genes in the *A. gambiae *genome [29].

Thioester-containing proteins (TEPs), initially identified in *D. melanogaster *[39], contain a sequence motif (GCGEQ) commonly found in members of the complement C3/α 2-macroglobulin superfamily. After cleavage activation, some TEPs use the metastable thioester bond between the cysteine and glutamine residues to covalently attach to pathogens and 'mark' them for clearance by phagocytosis [40]. One of the 15 TEPs in *Anopheles*, *Ag*-TEP1, plays a key role in the host response against *Plasmodium *infection and ten other *Ag*-TEPs are results of extensive gene duplications. This kind of family expansion did not happen in the beetle (or bee): *Tribolium *encodes four TEPs, perhaps for different physiological purposes. Our phylogenetic analysis supports the following orthologous relationships: *Tc*A-*Am*A-*Ag*13-*Dm*6, *Tc*B-*Am*B-*Ag*15-*Dm*3, and *Tc*C-*Am*C (Additional data file 6).

### Extracellular signal transduction and modulation

Similar to the alternative and lectin pathways for activation of human complements, insect plasma factors play critical roles in pathogen detection, signal relaying/tuning, and execution mechanisms. Serine proteinases (SPs) and their noncatalytic homologs (SPHs) are actively involved in these processes. Some SPs are robust enzymes that hydrolyze dietary proteins; others are delicate and specific - they cleave a single peptide bond in the protein substrates. The latter interact among themselves and with pathogen recognition proteins to mediate local responses against nonself. The specificity of such molecular interactions could be enhanced by SPHs, adaptor proteins that lack proteolytic activity due to substitution of the catalytic triad residues. SPs and SPHs constitute one of the largest protein families in insects [29,41,42]. We have identified 103 SP genes and 65 SPH genes in the *Tribolium *genome, 77 of which encode polypeptides with a SP or SP-like domain and other structural modules. These include thirty SPs and eighteen SPHs containing one or more regulatory clip domains. Clip-domain SPs, and occasionally clip-domain SPHs, act in the final steps of arthropod SP pathways [43]. Other recognition/regulation modules (for example, LDL_r_A, Sushi, CUB and CTL) also exist in long SPs (>300 residues), some of which act in the beginning steps of SP pathways.

**Figure 2:**
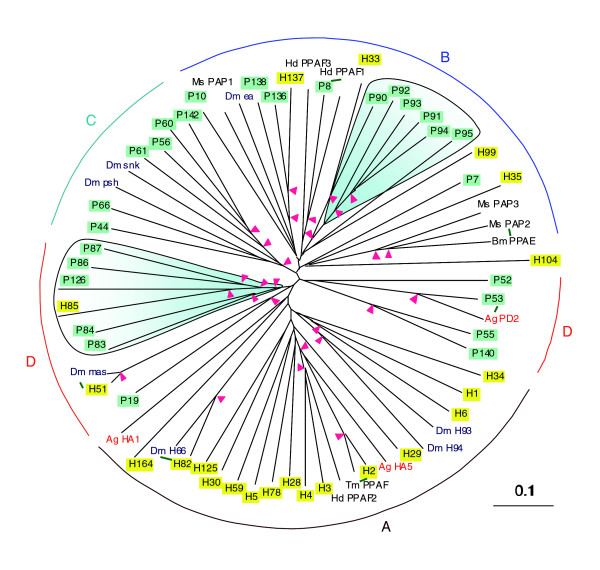
Expansion of the clip-domain family of SPs and SPHs in the *T. castaneum *genome. The catalytic and proteinase-like domains in the 49 *Tribolium *sequences are compared with those in 7 *Drosophila *(Dm), 3 *Anopheles *(Ag), 3 *Holotrichia *(Hd), 1 *Tenebrio *(Tm), 1 *Bombyx *(Bm) and 3 *Manduca *(Ms) SP-related proteins. The tree is divided to four clades (A to D). While clade A contains SPHs (yellow) only, the other three are mainly SPs (green). Region D, split into two parts, is intact when all the group D clip-domain proteins from *Drosophila *and *Anopheles *are included in the analysis (data not shown). Pink arrowheads at nodes indicate bootstrap values greater than 800 from 1,000 trials. The putative ortholog pairs are connected with green bars. Other than the shown ones (shaded blue, excluding SP126), there are four clusters of clip-domain SP/SPH genes in the genome: (SP)H1 through H6, (S)P7 through P10, H28 and H29, P135 through P139. Some of them (P9, P135 and P139) have no clip domain and, thus, are not shown in the figure.

*T. castaneum *clip-domain proteins are divided into four subfamilies (Figure 2). Even though the catalytic or proteinase-like domains used for comparison were similar in length and sequence, we found subfamily A is composed of SPHs solely whereas subfamilies B, C and D comprise SPs mainly. Apparently, it is easier for SPs to lose activity and become SPHs during evolution than for SPHs to regain catalytic activity. The four groups of SP-related genes may represent lineages derived from ancient evolutionary events since similar subfamilies also exist in *Anopheles *and *Drosophila*. Moreover, expansion of individual subfamilies must have occurred several times to account for the gene clusters observed in the *Tribolium *genome (Figure 2). Evidence for lineage-specific gene duplication and movement is also present in the mosquito and fly genomes [29,41]. Based on the results of genetic/biochemical analysis performed in other insects [14-16,19,44,45] and sequence similarity, we are able to predict the physiological functions for some *Tribolium *clip-domain SPs and SPHs during proPO activation and spätzle processing. For instance, *Tc-*SPH2, SPH3 or SPH4 (similar to *Hd*-PPAF2) may serve as a cofactor for *Tc*-SP7, SP8 or SP10 (putative proPO activating proteinases); *Tc*-SP44 or SP66 may function like *Drosophila *persephone [46]; *Tc*-SP136 or SP138 may activate spätzle precursors by limited proteolysis [44,45].

**Figure 3:**
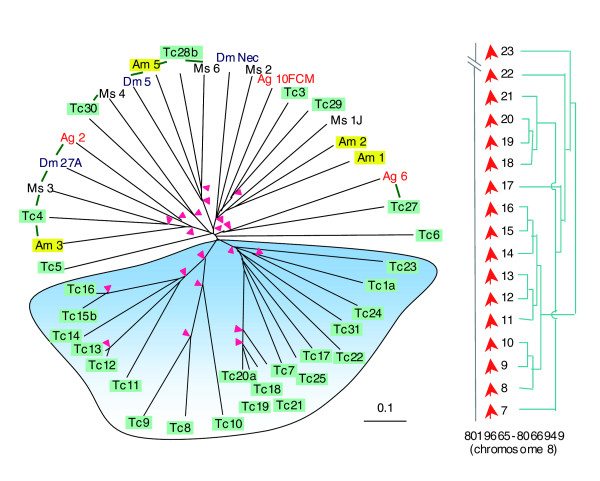
A major family expansion of *Tribolium *serpins and their phylogenetic relationships with the serpins from other insect species. The sequences of 29 *Tribolium *(Tc), 3 *Drosophila *(Dm), 3 *Anopheles *(Ag), 4 *Apis *(Am) and 5 *Manduca *(Ms) serpins are compared. *Tribolium *serpin2 (758 residues) and serpin26 (568 residues), much longer than a typical serpin (40-50 kDa), are excluded from the analysis. For simplicity, *Tribolium *serpins 1b, 15a, 20b and 28a are also eliminated because they are products of alternative splicing of the genes 1a, 15b, 20a and 28b, which differ only in the region coding for reactive site loop. As shown in the tree (left panel), extensive expansion gives rise to this group of highly similar genes (shaded blue) located in a small chromosomal region (right panel). Pink arrowheads at nodes denote bootstrap values greater than 800 for 1,000 trials. Putative 1:1, 1:1:1 or 1:1:1:1 orthologous relationship is indicated by green bars connecting the group members.

Most members of the serpin superfamily are irreversible inhibitors of SPs and, by forming covalent complexes with diffusing proteinases, they ensure a transient, focused defense response [47]. There are totally 31 serpin genes in *T. castaneum*, more than that in *D. melanogaster *(28), *A. gambiae *(14) or *A. mellifera *(7). This number increase is mainly caused by a recent family explosion at a specific genomic location - we have identified a cluster of 16 serpin genes in a small region of 50 kilobases on chromosome 8. These closely related genes constitute a single clade in the phylogenetic tree (Figure 3). Sequence divergence, especially in the reactive site loop region, is anticipated to alleviate the selection pressure imposed by the SP family expansion (Figure 2). Exon duplication and alternative splicing, found in 4 of the 31 serpin genes, also generate sequence diversity and inhibitory selectivity.

### Intracellular signal pathways and their regulation

**Figure 4:**
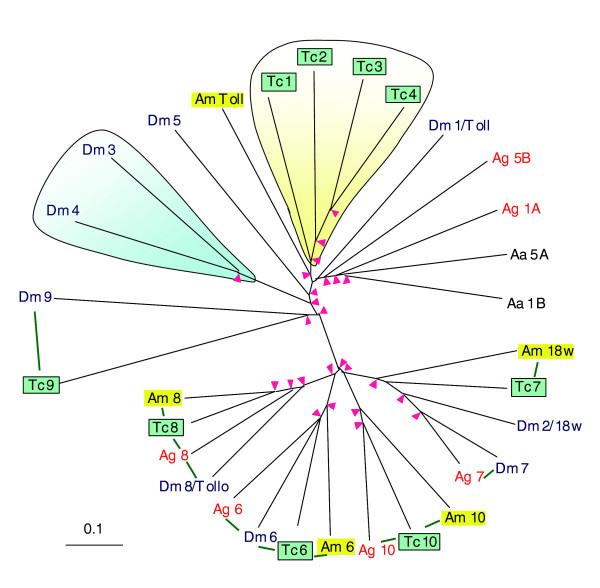
Phylogenetic relationships of Toll-like receptors from five insect species. The sequences of nine *Tribolium *(Tc), nine *Drosophila *(Dm), six *Anopheles *(Ag), five *Apis *(Am), and two *Aedes *(Aa) Toll-related proteins are compared. Species-specific family expansion is shaded yellow for *Tribolium *and blue for *Drosophila*. Nodes with pink arrowheads have bootstrap values exceeding 800 from 1,000 trials, and green lines connect putative orthologs with 1:1, 1:1:1 or 1:1:1:1 relationship. Note that *Tc*Toll-9 does not have a Toll/interleukin1 receptor domain.

*Drosophila *Toll is a transmembrane protein that binds spätzle and relays developmental and immune signals [48]. Resulting from ancient family expansion, a total of five spätzle homologs and eight Toll-like receptors are present in the fly. There are seven *Tribolium *genes coding for spätzle-like proteins, most of which have putative orthologs in *Drosophila *and *Anopheles *(Additional data file 7). Like their ligands, Toll-like proteins have also experienced major family expansion and sequence divergence. The receptors are separated into two clusters, with the fly and beetle Toll-9 located near the tree center (Figure 4). While Toll-6, -7, -8 and -10 from different insect species constitute tight orthologous groups in one cluster, lineage-specific gene duplications have given rise to *Drosophila *Toll-3 and -4, *Anopheles *Toll-1 and -5, as well as *Tribolium *Toll-1 through -4. Located on the same branch with *Drosophila *Toll, the four *Tribolium *receptors could play different yet complementary roles in the beetle defense and development. In addition, we have identified eight MD2-related genes in the beetle. Mammalian MD2, Toll-like receptor-4 and CD14 form a complex that recognizes lipopolysaccharides [49]. The *Anopheles *MD2-like receptor regulates the specificity of resistance against *Plasmodium berghei *[50].

**Figure 5:**
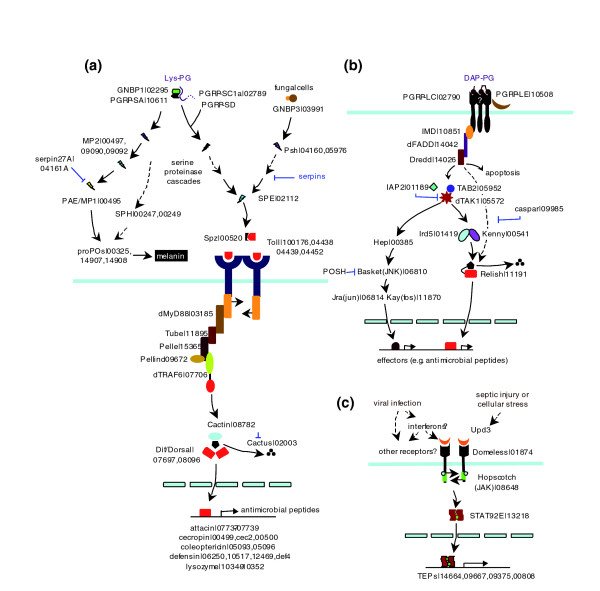
Schematic drawing of the immune signaling pathways in *Drosophila *and *Tribolium*. **(a) **Extracellular serine proteinase pathways for proPO and Spätzle activation as well as the intracellular Toll pathway for antimicrobial peptide production. **(b) **IMD pathway and JNK branch for induced synthesis of immune responsive effectors. **(c) **JAK-STAT pathway for transcription activation of defense genes (for example, TEPs). Components of the putative pathways from *T. castaneum *are predicted based on sequence similarity. The *Drosophila *gene names are followed by GLEAN numbers of their beetle orthologs (or paralogs in some cases).

Contrary to the ligand-receptor diversification, components of the intracellular pathway appear to be highly conserved in insects studied so far (Figure 5a). In *Drosophila*, multimerization of Toll receptors caused by spätzle binding leads to the association of dMyD88, Tube, Pelle, Pellino and dTRAF6 [51]. With 1:1 orthologs identified in the beetle (as well as the other insects with known genomes), we postulate that a similar protein complex also forms to phosphorylate a cactus-like molecule (Tc02003). The modified substrate protein then dissociates from its partner (Tc07697 or Tc0896), allowing the Rel transcription factors to translocate into the nucleus and activate effector genes (for example, antimicrobial peptides). Functional tests are required to verify the suggested roles of individual components during defense and development in the beetle.

The IMD pathway is critical for fighting certain Gram-negative bacteria in *Drosophila*. Upon recognition of diaminopimelate-peptidoglycan by PGRPs, the 'danger' signal is transduced into the cell through IMD (Figure 5b). IMD contains a death domain that recruits dFADD (dTAK1 activator) and Dredd (a caspase). Active dTAK1 is a protein kinase that triggers the JNK pathway (through Hep, Basket, Jra and Kay) and Relish phosphorylation (through Ird5 and Kenny). The presence of 1:1 orthologs in *T. castaneum *strongly suggests that IMD-mediated immunity is conserved in the beetle. Furthermore, the modulation of these pathways may also resemble each other - we have identified putative 1:1 orthologs of IAP2, Tab2 and caspar in the *Tribolium *genome (Figure 5b).

The transcription of *Drosophila *TEPs and some other immune molecules is under the control of the JAK-STAT pathway [52]. This pathway, triggered by a cytokine-like molecule, Upd3, promotes phagocytosis and participates in an antiviral response. Based on sequence similarity, we predict that the conserved signaling pathway in the beetle is composed of the orthologs of *Dm*-Domeless, Hopscotch and STAT92 (Figure 5c). However, we have not identified any ortholog of *Dm*-upd, upd2, or upd3, possibly due to high sequence variation in the cytokine-like proteins.

### Execution mechanisms

Phenoloxidases are copper-containing enzymes involved in multiple steps of several immune responses against pathogens and parasites (that is, clot reinforcement, melanin formation, ROS/RNS generation, and microbe killing) [53]. Synthesized and released as an inactive zymogen, proPO requires a SP cascade for its cleavage activation. SPHs and serpins ensure that the proteolytic activation occurs locally and transiently in response to infection. We have identified three proPO genes in the *Tribolium *genome, designated proPO1, 2 and 3. *Tc-*proPO2 and proPO3 are 98.8% identical in nucleotide sequence and 99.6% identical in amino acid sequence. In the aligned coding regions (2,052 nucleotides long), 21 of the 24 substitutions are synonymous, corresponding to 0.0102 changes/site. These two genes are 530 kb apart and their aligned intron regions are 88.5% identical. Using the relative rate of nucleotide substitutions derived from an analysis of *Drosophila *alcohol dehydrogenase genes [54], we estimate that *Tc*-proPO2 and *Tc*-proPO3 arose by gene duplication approximately 0.6 million years ago. The phylogenetic analysis suggests that such evolutionary events are sporadic for this family: the total numbers of proPO genes in different insect species did not change significantly, except for the malaria mosquito (Additional data file 8). Of the nine *Ag*-proPO genes, eight arose from gene expansion that occurred early in the mosquito lineage [29], some of which encode phenoloxidases for melanization.

Local production of free radicals is a critical component of the acute-phase oxidative defense, involving nitric oxide synthase, NADPH oxidase, peroxidase, phenoloxidase and other enzymes [53,55]. Due to the cytotoxicity of ROS and RNS, their conversion and concentrations must be tightly regulated by superoxide dismutases (SODs), glutathione oxidases (GTXs), catalases, thioredoxins, thioredoxin reductases, melanin intermediates, and certain metal ions. Changes in the free radical levels by gene mutation or knock-down affect the fecundity and antimalarial response of the mosquito [56]. We have annotated some of these genes in *Tribolium*, including peroxidases, GTXs, SODs, peroxiredoxins (TPXs) and catalases. *T. castaneum *GTX1-GTX2 and TPX2-TPX6 gene pairs are results of recent gene duplications, whereas several orthologous relationships have been identified in the SOD and TPX families in the phylogenetic analysis (Additional data file 9).

**Figure 6:**
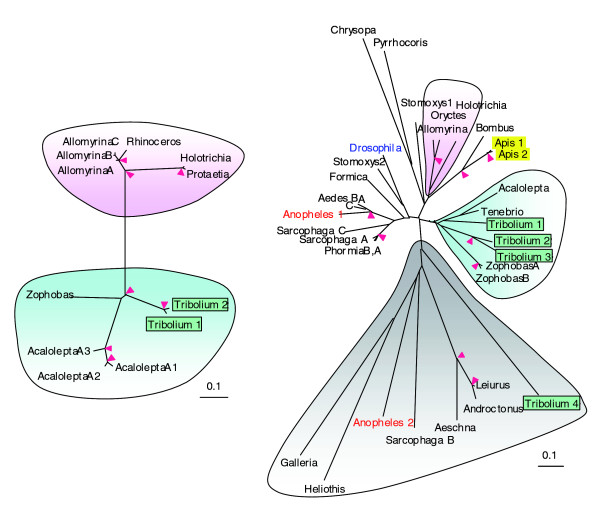
Evolutionary relationships of the coleoptericins (left panel) and defensins (right panel). The alignment of mature antimicrobial peptide sequences is used to build the phylogenetic trees on which their genus names are indicated. The beetle coleoptericins and defensins are divided into two subgroups (shaded blue and pink), whereas the more primitive defensins (shaded grey) are found in many arthropod species. Note that the blue clades include *Acalolepta*, *Tribolium *and *Zophobas *whereas the pink clades both contain *Allomyrina *and *Holotrichia*. Pink arrowheads at nodes denote bootstrap values greater than 800 from 1,000 trials. This analysis uses sequences from the orders of Coleoptera (*Acalolepta*, *Allomyrina*, *Holotrichia*, *Oryctes*, *Protaetia*, *Rhinoceros*, *Tenebrio*, *Tribolium*, *Zophobas*), Diptera (*Aedes*, *Anopheles*, *Drosophila*, *Phormia*, *Sarcophaga*, *Stomoxys*), Lepidoptera (*Galleria*, *Heliothis*), Hemiptera (*Pyrrhocoris*), Hymenoptera (*Apis*, *Bombus*, *Formic*), Neuroptera (*Chrysopa*), Ordonata (*Aeschna*) and Scopiones (*Androctonus*, *Leiurus*).

Coleopteran species have been explored at the biochemical level for various antimicrobial peptides (AMPs) [57]. While defensins are present in all insects studied, coleoptericins are related to the attacin/diptericin family of glycine-rich antibacterial peptides in lepidopteran and dipteran species [58]. Four defensin genes are detected in the *Tribolium *genome, three of which are found in a branch containing only coleopteran insects (Figure 6). *Tc*-defensin4 is in a miscellaneous group containing Odonata, Lepidoptera and Arachnida species. Interestingly, defensins of three other coleopteran insects are in the same branch with the hymenopteran ones. Like the beetle defensins, coleoptericins belong to two phylogenetic groups, with the same separation of species in each group.

With the genome sequence available, we are able to use the other AMP sequences to identify homologous genes that are not specified in beetles. Cecropins were mostly identified in moths and flies - there was only one report on cecropin from a coleopteran species, *Acalolepta luxuriosa *[11]. In *Tribolium*, we find a single close homolog of the *Acalolepta *cecropin, although a frame shift in a run of seven adenosines indicate that this is a pseudogene (*Tc*00499). Closely linked to *Tc*00499 on chromosome 2 are two genes that encode cecropin-related peptides of unusual structure, with proline- and tyrosine-rich carboxy-terminal extensions (*Tc*-cecropin2 and Tc00500). These observations indicate that cecropins may widely exist in beetles. Attacins were found only in lepidopteran and dipteran species. We have identified a cluster of three attacin genes (*Tc*07737-07739) on *Tribolium *chromosome 4. Although we failed to identify a Drosomycin homolog in the beetle, our search resulted in a low-score hit of a cysteine-rich sequence. The corresponding gene (*Tc*11324) encodes a 104 residue polypeptide containing 2 whey acidic protein motifs. While mammalian proteins with this motif possess antibacterial activities [59], expression and biochemical analyses are needed to test if the *Tribolium *protein has a similar function. Due to the presence of species-specific AMPs and severe sequence diversity of these molecules, our homology-based search has probably missed some AMP genes. Should there be a thorough exploration by sequence similarity, biochemical separation and activity assays (not only against Gram-positive and Gram-negative bacteria, but also against yeasts and filamentous fungi), we expect the total number of AMPs (currently 12) in *T. castaneum *may approach that (20) in *D. melanogaster*. In addition to these, we have found a cluster of four lysozyme genes in the *Tribolium *genome (Additional data file 10). Similar but independent family growths have occurred in different insect groups, giving rise to thirteen such genes in *Drosophila*, eight in *Anopheles*, three in *Apis*, and four in *Tribolium*.

**Figure 7:**
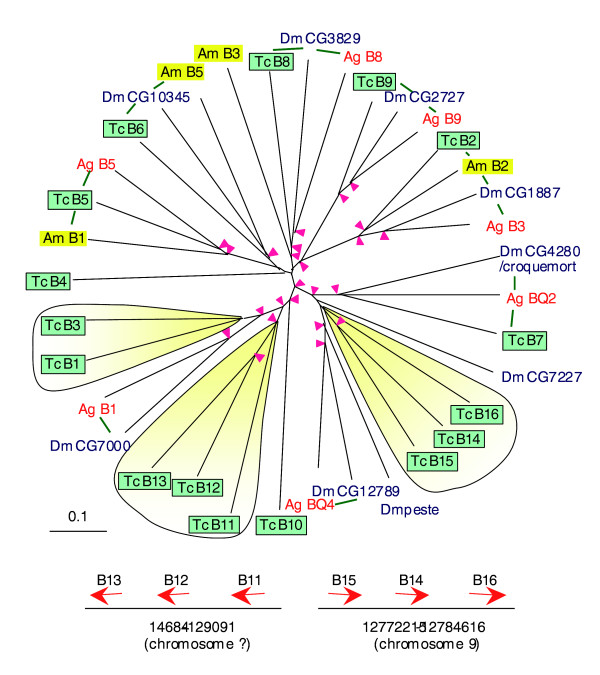
Phylogenetic analysis of class B SRs (SR-Bs). The aligned central parts, including the CD36 domain, of sixteen *Tribolium *(Tc), eight *Drosophila *(Dm), eight *Anopheles *(Ag) and three *Apis *(Am) SR-B sequences are used for building the unrooted tree (upper panel). For simplicity, the other members of class B SRs from *Drosophila *(seven) and *Anopheles *(four) are not included in this analysis. Lineage-specific expansion (shaded yellow) is confirmed in the complete tree that includes all SR-Bs from the four species. The expansion is consistent with their chromosomal locations (lower panel). Pink arrowheads indicate nodes with bootstrap values exceeding 800 (from 1,000 trials), whereas green bars connect the putative orthologs with 1:1, 1:1:1 or 1:1:1:1 relationship.

Cellular responses (that is, phagocytosis, nodulation and encapsulation) play key roles in the insect innate immunity [60]. In the past few years, breakthroughs have been made in the molecular dissection of these processes [61]. *Drosophila *Peste, Eater, scavenger receptor (SR)-CI, Dscam, TEPs, and PGRP-SC1a seem to be implicated in the phagocytosis. Multiple SR-B genes are present in the *Tribolium *(16), *Drosophila *(12) and *Anopheles *(16) genomes, indicative of important functions of the subfamily. A phylogenetic analysis of the SR-Bs (Figure 7) demonstrates that nearly half of the members arose from ancient gene duplication events - we can easily identify orthologs from different insect species. More recent family expansions in the mosquito [29] and beetle account for the other half of the subfamily. There are two SR-B gene clusters in the *Tribolium *genome, one of which (*Tc*SR-B14, -B15 and -B16) is located in the same branch containing *Dm-*peste. In addition to SR-Bs, *Drosophila *Nimrods are also involved in cellular responses [62]. The plasmatocyte-specific NimC1 directly participates in the phagocytosis of bacteria. For *Tribolium*, all three subclasses are represented: NimA, NimB and NimC, just like in the fly, mosquito and bee. However, unlike the other insects, the syntenic relationship is broken up in the beetle NimC homologs: the two NimC paralogs (*Tc*02053 and *Tc*15258) are not closely linked to the NimA and NimB homologs (*Tc*11427 and *Tc*11428). In the other insects, the order of nimA, nimB and nimC genes is well conserved.

### Expression analysis

**Figure 8:**
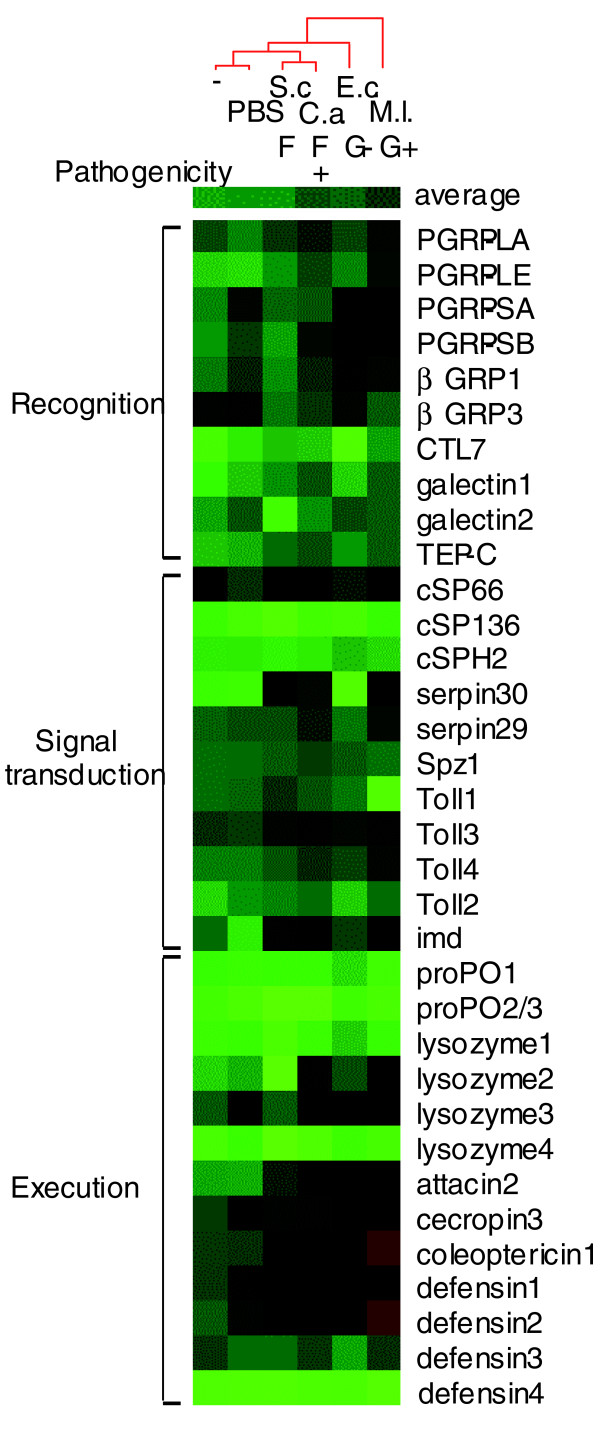
Real-time PCR analysis of expression of *Tribolium *immunity-related genes in adults 24 h after injections of *M. luteus *(*M.l*.), *E. coli *(*E.c*.), *C. albicans *(*C.a*.), *S. cerevisiae *(*S.c*.), or phosphate-buffered saline (PBS). Uninjured insects (-) were used as another negative control. With green, black and red colors representing low, intermediate and high transcript levels, respectively, relative mRNA abundances were used to cluster samples by average-linker clustering.

One characteristic of the innate immune system is that some of its components are transcriptionally up-regulated after a microbial challenge. To acquire evidence that the genes we annotated are involved in defense responses, we have exposed the adult beetles to *E. coli*, *Micrococcus luteus*, *Candida albicans *or *Saccharomyces cerevisiae *cells and isolated total RNA from the control and treated insects for expression analysis. Real-time PCR experiments indicated that transcript levels of some genes dramatically changed (Figure 8). *Tc*PGRP-SA and *Tc*PGRP-SB mRNA became more abundant after the bacterial infection, whereas the increase was much less significant for *Tc*PGRP-LA, -LE, galectin1 or TEP-C after the *C. albicans *or *M. luteus *treatment. Following the Gram-positive bacterial or fungal challenge, we detected some elevations in *Tc*-cSP66, serpin29 and serpin30 transcripts.

Transcriptional regulation is not limited to pattern recognition molecules or extracellular signal mediators/modulators: we detected differential expression of ligand and their receptors (for example, *Tc*-spätzle1, Toll-1 through Toll-4, and IMD). mRNA level changes for the latter genes were small except for IMD (Figure 8). Toll-3 and Toll-4 induction after the *C. albicans *or *M. luteus *challenge was apparent, although not as notable as IMD. The subtle changes in Toll-1 transcript levels were somewhat different from those of Toll-2, -3 and -4, indicating that there could be functional differences and overlaps in antimicrobial responses for these closely related receptors (Figure 4).

We have also examined genes whose products are plasma proteins directly involved in microbe immobilization or killing. The transcripts of *Tc*-proPOs, lysozyme1 or lysozyme4 did not significantly change when compared with the controls, whereas those of *Tc*-lysozyme2 and 3 increased remarkably (Figure 8). The most dramatic increase in mRNA levels occurred in the AMP group of effector molecules, including *Tc*-attacin2, cecropin3, coleoptericin1, defensin1, and defensin2.

Cluster analysis of the expression patterns has revealed several trends of the transcriptional control of these immune genes. Buffer injected and uninjured adults form one cluster with the lowest mRNA levels, whereas *E. coli*- and *S. cerevisiae*-treated insects have the next higher level of overall gene expression (Figure 8). The yeast-injected beetles, instead of grouping with *E. coli*-treated insects, are found in the same cluster with *C. albicans-*challenged adults. Interestingly, immune responses toward the opportunistic fungal pathogen are greater than those toward *S. cerevisiae*, an environmental non-pathogen present in the diet. The responses toward *M. luteus *and *C. albicans *were significantly stronger than those towards *E. coli*, implying that the Toll pathway triggered by the Gram-positive bacteria and filamentous fungi more effectively up-regulated target gene expression than the IMD pathway did, which may be activated by the Gram-negative bacterial infection (Figure 5).

## Conclusion

Through this comparative genome analysis, we have provided evidence in the red flour beetle for the functional conservation of intracellular immune signaling pathways (Toll, IMD and JAK/SAT) and for the evolutionary diversification of over 20 families of proteins (for example, PGRPs, clip-domain proteins, serpins, Toll-related receptors, antimicrobial proteins and scavenger receptors) involved in different mechanisms of insect defense against infection. The observed differences in conservation are likely related to distinct needs for specific molecular interactions and changes in microorganisms encountered by the host insects. For instance, *Drosophila *Myd88, Tube, Pelle, Pellino and TRAF, which form a macromolecular complex with the Toll/interleukin 1 receptor domain (Figure 5), have 1:1 orthologs in *Anopheles*, *Apis *and *Tribolium*. In contrast, family expansion and sequence divergence in the PGRP and AMP families are perhaps important for specific recognition and effective elimination of evolving pathogens.

The summary of putative immune gene counts, families and functions (Additional data file 11) suggests that *T. castaneum *has a more general defense than *A. gambiae *does. While this system is critical for the survival of this beetle, we are unclear whether or not it correlates with the prosperity of coleopteran insects. Drastic lineage-specific expansions seem sporadic and, in most cases, *Tribolium *paralog counts are lower than those of *Anopheles *or *Drosophila *(but are considerably higher than of *Apis*). The only exceptions are the clip-domain SP/SPH and serpin families: 48, 41 and 37 proteinase-related genes and 31, 14 and 28 inhibitor genes are present in the beetle, mosquito and flies, respectively. Because clip-domain SPs are often regulated by serpins, positive selection may have played a role in the converted evolution of both families and in the maintenance of homeostasis.

This comparative analysis has also uncovered interesting genes and gene families for future research. For instance, the existence of a 1:1 ortholog of *Drosophila *PGRP-LE in *Tribolium *(but not in *Anopheles *or *Apis*) may allow us to test whether or not *Tc*PGRP-LE has a similar function. It can be interesting to explore the molecular mechanisms and evolutionary pathways of the large serpin and SP gene clusters in the beetle. The presence of *Tc*Toll-1 through -4 and subtle changes in their mRNA levels after immune challenges call for detailed analysis of their transcriptional regulation and physiological functions. Of course, the proposed extracellular and intracellular signaling pathways need to be tested, even though we have confidence in their general structures. The possible AMP function of *Tc*11324, which contains two whey acidic protein motifs, needs to be established experimentally.

It is noteworthy that the functions of *Tribolium *immunity-related genes are mostly assumed based on sequence similarity to studied proteins in *Drosophila *or other insect species. Functional analyses using the strong reverse genetic techniques available in *Tribolium *are necessary to test the hypotheses. Nevertheless, the framework of information established in this work should help clarify immune functions in an important agricultural pest from the most diverse insect order and a species that can serve as a tractable model for an innate immune system more generally.

## Materials and methods

### Database search and sequence annotation

Known defense proteins from other insects were used as queries to perform BLASTP searches of *Tcastaneum *Glean Predictions (2005.10.11) [63]. Protein sequences with E-values lower than 0.1 were listed, and every 5th sequence was retrieved for use as a query for another round of search. Based on the combined lists, respective protein sequences were retrieved, compiled in the order of ascending E-values, and improved by two methods. Firstly, *Tcastaneum *ESTs (2005.9.20) at the same HGSC site were searched with the corresponding nucleotide sequences to identify possible cDNA clones. The EST sequences were assembled using CAP3 [64] and the resulting contigs were used in pairwise comparison [65] to validate the gene predictions. Secondly, retrieved protein sequences were analyzed by CDART [66], PROSITE [67], and SMART [68] to detect conserved domain structures required for specific functions. Necessary changes were made after each step to improve the original predictions. Chromosomal location and exon-intron boundaries for each annotated sequence were acquired from Genboree [69]. To locate orthologs not identified by BLASTP, *Tribolium *Genome Assembly 2.0 [70] was searched using TBLASTN. The hits detected were analyzed using multiple gene prediction tools Genescan and Genemark [71,72]. All curated sequences then were deposited in the annotation database [73] as a part of *Tribolium *Genome Assembly 2.0.

### Phylogenetic analyses

Unless otherwise specified, full-length *Tribolium *sequences were aligned with their homologs from other insects, including *D. melanogaster*, *A. gambiae *and *A. mellifera*. The sequences were retrieved from NCBI [74], Flybase [75], or Ensembl [76]. Multiple sequence alignments were carried out using ClustalX [77] and Blosum series of weight matrices [78]. Phylogenetic trees were constructed based on algorithm of neighbor-joining using PHYLIP [79] or maximum-parsimony using PAUP [80]. The divergence time of *Tc-*proPO2 and proPO3 were calculated using the rate of 1.7 × 10^-8 ^synonymous substitutions/nucleotide/year derived from the *Drosophila *species [54].

### Gene expression analysis

To study pathogen-induced gene expression, adult red flour beetles (approximately 240 per group) were pricked at the ventral thorax with needles dipped in sterile phosphate-buffered saline or the buffer containing concentrated live *E. coli*, *M. luteus*, *C. albicans *or *S. cerevisiae *cells. Uninjured and aseptically injured insects were employed as controls. Total RNA samples were extracted from the control and challenged insects (approximately 160 per group) 24 h later, using Micro-to-mid RNA Purification System (Invitrogen, Carlsbad, CA, USA). After DNA removal, each RNA sample (1.0-3.4 μg), oligo(dT) (0.5 μg, 1 μl) and dNTPs (10 mM each, 1 μl) were mixed with diethyl pyrocarbonate-treated H_2_O in a final volume of 12 μl, and denatured at 65°C for 5 minutes. First strand cDNA was synthesized for 50 minutes at 42°C using SuperScript Reverse Transcriptase (200 U/μl, 1 μl; Invitrogen) mixed with 5 × buffer (4 μl), 0.1 M dithiothreitol (2 μl), RNase OUT (40 U/μl, 1 μl; Invitrogen) and the denatured RNA sample (12 μl). Specific primer pairs were designed for a total of 35 immunity-related genes (Additional data file 12) using Primer 3 [81] with annealing temperatures of 59.5-60.5°C and expected product sizes of 80-150 bp. Each primer pair was located in adjacent exons flanking an intron. Real-time PCR was performed in parallel reactions on 96-well microtiter plates using Taq DNA polymerase (1 U; Roche Applied Sciences, Indianapolis, IN, USA), 1 × buffer, 1 mM dNTP mix, 2 mM MgCl_2_, 0.2 μM primers, 1 × SYBR-Green I dye (Applied Biosystems, Foster City, CA, USA) and 10 nM fluorescein. Amplifications were enacted on an iCycler thermal cycler (Bio-Rad, Hercules, CA, USA) with a profile of 95°C for 5 minutes followed by 40 cycles of 94°C for 20 s, 60°C for 30 s, 72°C for 60 s and 78°C for 20 s [82]. SYBR green fluorescence was measured during the 78°C step in each cycle and the cycle numbers for each target and control gene were recorded when the fluorescence passed a predetermined threshold. Proper dissociation and correct size of the products were examined by melting curve analysis and agarose gel electrophoresis, respectively. The real-time PCR was repeated twice and, in each of the three experimental replicates, the transcripts were normalized relative to the levels of *Tribolium *ribosomal protein S3. Averaged transcript abundance values (Ct_control _- Ct_target_) were then compared across genes and samples using average-linking clustering (Cluster 3.0) and visualized using TreeView [83].

## Abbreviations

β GRP, β-1,3-glucan-recognition protein; AMP, antimicrobial peptide; CTL, C-type lectin; FREP, fibrinogen-related protein; GNBP, Gram-negative binding protein; GTX, glutathione oxidase; PGRP, peptidoglycan recognition protein; PPAF, proPO activating factor; proPO, prophenoloxidase; RNS, reactive nitrogen species; ROS, reactive oxygen species; SOD, superoxide dismutase; SP, serine proteinase; SPH, noncatalytic serine proteinase homolog; SR, scavenger receptor; TEP, thioester-containing protein; TPX, peroxiredoxin.

## Authors' contributions

Zhen Zou: study design; data collection, analysis and deposition; annotation of clip-domain SPs/SPHs, serpins, spätzles, SRs and others; Toll and Imd pathways. Jay Evans: RT-PCR analysis; GNBPs and PGRPs. Zhiqiang Lu: C-type lectins, galectins, TEPs and JAK/STAT pathway. Picheng Zhao: Toll-like receptors, caspases and ROS/RNS production. Michael Williams and Dan Hultmark: FREPs, Nimrods, PGRPs and cecropins. Charles Hetru and Niranji Sumathipala: antimicrobial peptides and lysozymes. Haobo Jiang: study design; data analysis and interpretation; annotation of clip-domain SPs/SPHs; manuscript writing.

## Additional data files

The following additional data are available with the online version of this paper. Additional data file 1 is a table listing immunity-related genes in *T. castaneum*. Additional data file 2 is a figure showing sequence alignments of βGRPs and GNBPs. Additional data file 3 is a figure showing sequence alignments of CTLs. Additional data file 4 is a figure showing sequence alignments of galectins. Additional data file 5 is a figure showing sequence alignments of FREPs. Additional data file 6 is a figure showing sequence alignments of TEPs. Additional data file 7 is a figure showing sequence alignments of Spätzle-related proteins. Additional data file 8 is a figure showing sequence alignments of proPOs. Additional data file 9 is a figure showing sequences of GTX, SOD and TPX. Additional data file 10 is a figure showing sequence alignments of lysozymes. Additional data file 11 is a table listing functions, families, and counts of putative defense proteins from *D. melanogaster*, *A. gambiae*, *A. mellifera *and *T. castaneum*. Additional data file 12 is a table listing oligonucleotide primers used in expression analysis by real-time PCR.

## Supplementary Material

Additional data file 1 Immunity-related genes in *T. castaneum* Click here for file

Additional data file 2 The sequences of three *Tribolium *(Tc), three *Drosophila *(Dm), two *Apis *(Am), six *Anopheles *(Ag) and two *Bombyx *βGRPs/GNBPs are aligned with *Bacillus circulans *(Bc) β-1,3-glucanase A1 as an outgroup. There was a family expansion in the lineage of *A. gambiae*. Pink arrowheads indicate nodes with bootstrap values greater than 800 from 1,000 trials, and the dashed line marks the outgroup. Click here for file

Additional data file 3 The sequences of sixteen *Tribolium *(Tc), ten *Drosophila *(Dm), eight *Anopheles *(Ag) and eight *Apis *(Am) sequences are aligned. *Tc*CTL3 (that is, *Tc *3) contains two carbohydrate recognition domains and the first one is used for comparison. Different CTL subfamilies (GA, galactose; MA, mannose) are indicated, with the predicted orthologous groups marked by blue dots (for 1:1, 1:1:1 and 1:1:1:1 relationships). Pink arrowheads indicate nodes with significant bootstrap values (>800 of 1,000 trials). Note that many *Dm*- and *Ag*-CTLs, not included in this analysis, are results of major lineage-specific expansions [29]. Click here for file

Additional data file 4 The amino acid sequences from three *Tribolium *(Tc), seven *Drosophila *(Dm), seven *Anopheles *(Ag), two *Apis *(Am) and one *Phlebotomus *(Pp) galectins are examined. The phylogenetic tree, derived from the aligned sequences, shows family expansions in *Anopheles *(pink) and *Drosophila *(blue). Pink arrowheads at nodes denote bootstrap values greater than 800 from 1,000 trials. Green lines connect the putative orthologous pairs or trio. Click here for file

Additional data file 5 The sequences of seven *Tribolium *(Tc), fourteen *Drosophila *(Dm), nine *Anopheles *(Ag), nine *Aedes *(Aa) and one *Apis *(Am) FREPs are aligned for constructing this unrooted tree. For simplicity, other family members from *Drosophila*, *Anopheles *and *Aedes *are excluded from the analysis. Lineage-specific expansions (shaded yellow for *Tribolium*, blue for *Drosophila *and pink for *Anopheles*) are confirmed in the complete tree that includes all FREPs from these four species (data not shown). Nodes with pink arrowheads have bootstrap values exceeding 800 in 1,000 trials. Green bars connect the putative orthologs with 1:1 or 1:1:1 relationship. The chromosomal locations (lower corner) of *Tribolium *FREP-1 through -4 are shown. Click here for file

Additional data file 6 The sequences of four *Tribolium *(Tc), six *Drosophila *(Dm), fifteen *Anopheles *(Ag) and three *Apis *(Am) TEPs are aligned. Lineage-specific family expansions are indicated with color shades (blue for *Drosophila *and pink for *Anopheles*). Pink arrowheads at nodes denote bootstrap values greater than 800 for 1,000 trials, and green bars link the predicted 1:1 and 1:1:1:1 orthologs. Click here for file

Additional data file 7 The amino acid sequences of seven *Tribolium *(Tc), six *Drosophila *(Dm), five *Anopheles *(Ag), two *Apis *(Am) and one *Bombyx *(Bm) spätzles are aligned for building the unrooted tree. Pink arrowheads indicate nodes with significant bootstrap values (>800 of 1,000 trials), and green bars connect the putative orthologous pairs or trios. Click here for file

Additional data file 8 The entire sequences of *Tribolium *(Tc), *Tenebrio *(Tm), *Holotrichia *(Hd), and *Drosophila *(Dm), *Anopheles *(Ag), *Apis *(Am), *Bombyx *(Bm) and *Manduca *(Ms) proPOs are compared. *Tribolium *proPO3, >99% identical in amino acid sequence to *Tc*-proPO2, is not included in the analysis. The phylogenetic tree, derived from the multiple sequence alignment, shows the extensive family expansion (shaded pink) in the malaria mosquito. Pink arrowheads point to nodes with high bootstrap values (>800 from 1,000 trials), and green lines link the predicted 1:1 or 1:1:1 orthologs. Click here for file

Additional data file 9 **(a) **GTX, **(b) **SOD and **(c) **TPX. The *Tribolium *(Tc), *Drosophila *(Dm), *Anopheles *(Ag) and *Apis *sequences are studied. As shown in the trees, duplication and divergence have given rise to gene clusters (shaded yellow for *Tribolium *and blue for *Drosophila*). Pink arrowheads denote nodes with high bootstrap values (>800 in 1,000 trials), whereas green lines connect the putative orthologs with 1:1, 1:1:1 or 1:1:1:1 relationships. Click here for file

Additional data file 10 The sequences of four *Tribolium *(Tc), twelve *Drosophila *(Dm), five *Anopheles *(Ag), one *Bombyx *(Bm), one *Manduca *(Ms), and two *Apis *(Am) lysozymes are aligned and used to derive the tree (upper panel). Lineage-specific expansion (shaded in different colors) occurs quite extensively in this family of enzymes. For instance, four *Tribolium *lysozyme genes are found as a gene cluster (lower panel) at the same genomic location. Pink arrowheads at nodes indicate bootstrap values greater than 800 from 1,000 trials. A green bar links the putative orthologous pair. Click here for file

Additional data file 11 Functions, families, and counts of putative defense proteins from *D. melanogaster*, *A. gambiae*, *A. mellifera *and *T. castaneum* Click here for file

Additional data file 12 Oligonucleotide primers used in expression analysis by real-time PCR Click here for file
