# Supplementary material for: Comparative genomic analysis of the Tribolium immune system
Source: Genome Biol. 2007 Aug 29;8(8):R177. doi: 10.1186/gb-2007-8-8-r177 (PMC2375007; doi:10.1186/gb-2007-8-8-r177)
Supplement: Additional data file 1 — Immunity-related genes in T. castaneum [file gb-2007-8-8-r177-S1.doc]

**Supplemental Materials**

**Table S1. Immunity-related genes in *Tribolium castaneum***

| **Gene ID** | **Gene name** | **Gene family** | **Length (aa)** | **Putative *Dm* ortholog** | **Chromosome** |
| --- | --- | --- | --- | --- | --- |

GLEAN_02546 PGRP-LD PGRP 248 3

GLEAN_02789 PGRP-LA PGRP 343 3

GLEAN_02790 PGRP-LC PGRP 323 3

GLEAN_10508 PGRP-LE PGRP 324 PGRP-LE unknown

GLEAN_10611 PGRP-SA PGRP 195 PGRP-SA unknown

GLEAN_13620 PGRP-SB PGRP 188 5

GLEAN_15689 PGRP-LB PGRP 207 PGRP-LB 6

GLEAN_02295 βGRP1 βGRP/GNBP 378 unknown

GLEAN_03991 βGRP3 βGRP/GNBP 481 3

GLEAN_11529 βGRP2 βGRP/GNBP 441 unknown

GLEAN_06978 CTL1 C-type lectin 325 CG9134 unknown

GLEAN_14184 CTL2 C-type lectin 305 5

GLEAN_10898 CTL3 C-type lectin 402 CG6014 unknown

GLEAN_10947 CTL4 C-type lectin 295 unknown

GLEAN_10419 CTL5 C-type lectin 217 CG6055 unknown

GLEAN_03708 CTL6 C-type lectin 213 CG4115 3

GLEAN_14053 CTL7 C-type lectin 279 5

GLEAN_10412 CTL8 C-type lectin 309 CG14866 unknown

GLEAN_14328 CTL9 C-type lectin 679 CG15765 5

GLEAN_03135 CTL10 C-type lectin 564 3

GLEAN_03136 CTL11 C-type lectin 2033 3

GLEAN_13632 CTL12 C-type lectin 1180 contactin 5

GLEAN_13911 CTL13 C-type lectin 231 CG3244 5

GLEAN_00871 CTL14 C-type lectin 3631 SP1070 2

GLEAN_02811 CTL15 C-type lectin 1259 CG1500 (fw) 3

GLEAN_02984 CTL16 C-type lectin 860 CG9095 3

GLEAN_07619 GALE1 galectin 361 galectin (CG11372) 4

GLEAN_11871 GALE2 galectin 354 9

GLEAN_14802 GALE3 galectin 1235 5

GLEAN_03194 FREP5 fibrinogen-like 475 scabrous 3

GLEAN_03276 FREP1 fibrinogen-like 469 3

GLEAN_03277 FREP2 fibrinogen-like 263 3

GLEAN_03278 FREP3 fibrinogen-like 281 3

GLEAN_03294 FREP4 fibrinogen-like 186 3

GLEAN_04004 FREP6 fibrinogen-like 638 3

GLEAN_04864 FREP7 fibrinogen-like 456 CG9593 1=x

GLEAN_14664 TEP-B TEP 1475 tep3 5

GLEAN_09667 TEP-C TEP 1454 7

GLEAN_09375 TEP-A TEP 1766 mcr (tep6) 7

GLEAN_00808 TEP-D TEP 1718 2

GLEAN_00246 H1 cSPH 376 CG5390 2

GLEAN_00247 H2 cSPH 401 CG5390 2

GLEAN_00248 H3 cSPH 406 CG5390 2

GLEAN_00249 H4 cSPH 425 CG5390 2

GLEAN_00250 H5 cSPH 322 CG5390 2

GLEAN_00252 H6 cSPH 371 CG8738 2

GLEAN_00494 P7 cSP 382 CG9733 2

GLEAN_00495 P8 cSP 373 CG3066 2

GLEAN_00496 P9 SP 274 2

GLEAN_00497 P10 cSP 374 CG1102 2

GLEAN_00545 P11 SP 286 CG18735 2

GLEAN_00546 P12 SP 325 CG11836 2

GLEAN_00547 P13 SP 306 CG4914 2

GLEAN_00548 H14 SPH 296 2

GLEAN_00550 P15 SP 346 2

GLEAN_00635 P16 SP 287 CG14892 2

GLEAN_00740 H17 SPH 290 2

GLEAN_00829 H18 SPH 394 2

GLEAN_00870 P19 SP 1640 nudel 2

GLEAN_00989A P20 SP 253 2

GLEAN_00989B P21 SP 273 2

GLEAN_01023 P22 SP 324 CG4386 2

GLEAN_01157 P23 SP 271 CG30025 2

GLEAN_01158A P24 SP 254 CG31954 2

GLEAN_01158B P25 SP 268 alphaTry 2

GLEAN_01158C P26 SP 273 2

GLEAN_01159 P27 SP 250 Try29F 2

GLEAN_01300 H28 cSPH 347 CG5390 2

GLEAN_01301 H29 cSPH 355 CG5390 2

GLEAN_01908 H30 cSPH 327 CG5390 unknown

GLEAN_01946 H31 SPH 300 unknown

GLEAN_02061 P32 SP 295 CG11843 unknown

GLEAN_02112 H33 cSPH 344 CG16705 unknown

GLEAN_02150 H34 cSPH 313 CG5390 unknown

GLEAN_02193 H35 cSPH 333 CG3066 unknown

GLEAN_02659 P36 SP 207 Sb 3

GLEAN_02766 P37 SP 276 CG10472 3

GLEAN_02767 P38 SP 274 CG10472 3

GLEAN_02768 P39 SP 269 3

GLEAN_02785 P40 SP 259 CG16749 3

GLEAN_02786 P41 SP 255 CG16749 3

GLEAN_03081 P42 SP 306 CG6865 3

GLEAN_04084 P43 SP 284 unknown

GLEAN_04160 P44 cSP 506 snake unknown

GLEAN_04418 P45 SP 278 alphaTry 1=x

GLEAN_04523 P46 SP 653 CG31217 1=x

GLEAN_04524A H47 SPH 384 1=x

GLEAN_04524B H48 SPH 281 1=x

GLEAN_04524C H49 SPH 321 1=x

GLEAN_04535 P50 SP 636 CG31217 1=x

GLEAN_04622 H51 cSPH 726 masquerade 1=x

GLEAN_04624 P52 cSP 386 CG9372 1=x

GLEAN_04635 P53 cSP 498 CG31728 1=x

GLEAN_04654 P54 SP 1247 corin 1=x

GLEAN_04770 P55 cSP 372 CG1299 1=x

GLEAN_04863 P56 cSP 355 snake 1=x

GLEAN_04900 H57 SPH 364 CG13318 1=x

GLEAN_04937 P58 SP 193 1=x

GLEAN_04957 H59 cSPH 350 CG5390 1=x

GLEAN_05130 P60 cSP 364 snake unknown

GLEAN_05230 P61 cSP 355 snake unknown

GLEAN_05327 P62 SP 592 CG30106 8

GLEAN_05635 H63 SPH 965 8

GLEAN_05908 H64 SPH 265 8

GLEAN_05925 H65 SPH 262 8

GLEAN_05976 P66 cSP 375 CG6361 8

GLEAN_06026 P67 SP 252 8

GLEAN_06033 P68 SP 561 8

GLEAN_06034 P69 SP 477 8

GLEAN_06246 H70 SPH 251 8

GLEAN_06247 H71 SPH 253 8

GLEAN_06268 P72 SP 274 8

GLEAN_06269 H73 SPH 228 8

GLEAN_06424 P74 SP 288 8

GLEAN_06438 P75 SP 260 kappaTry 8

GLEAN_07017 P76 SP 258 Try29F 4

GLEAN_07019 P77 SP 251 Try29F 4

GLEAN_07026 H78 cSPH 375 CG5390 4

GLEAN_08267 P79 SP 261 4

GLEAN_08504 P80 SP 261 Try29F 4

GLEAN_08505 H81 SPH 387 4

GLEAN_08554 H82 cSPH 367 CG4998 unknown

GLEAN_08653 P83 cSP 687 CG8170 7

GLEAN_08657 P84 cSP 385 CG13744 7

GLEAN_08658A H85 cSPH 448 CG8172 7

GLEAN_08658B P86 cSP 843 CG11824 7

GLEAN_08659 P87 cSP 981 CG8213 7

GLEAN_08930 H88 SPH 248 7

GLEAN_08931 H89 SPH 390 7

GLEAN_09089 P90 cSP 371 easter 7

GLEAN_09090 P91 cSP 393 CG1102 7

GLEAN_09091 P92 cSP 373 CG5896 7

GLEAN_09092 P93 cSP 375 CG1102 7

GLEAN_09093 P94 cSP 379 CG5896 7

GLEAN_09094 P95 cSP 360 CG5896 7

GLEAN_09602 P96 SP 277 CG10472 7

GLEAN_09752A P97 SP 307 7

GLEAN_09752B P98 SP 403 CG30375 7

GLEAN_10076 H99 cSPH 368 CG17572 7

GLEAN_10781 P100 SP 645 corin unknown

GLEAN_10904 H101 SPH 266 unknown

GLEAN_10905A H102 SPH 339 unknown

GLEAN_10905B H103 SPH 261 unknown

GLEAN_10906 H104 cSPH 333 unknown

GLEAN_10907 H105 SPH 251 unknown

GLEAN_10908 H106 SPH 262 unknown

GLEAN_10909 H107 SPH 256 unknown

GLEAN_10910 H108 SPH 177 unknown

GLEAN_10911 H109 SPH 268 unknown

GLEAN_10927 H110 SPH 265 CG10477 unknown

GLEAN_10929 H111 SPH 258 unknown

GLEAN_10930 H112 SPH 262 unknown

GLEAN_10932 H113 SPH 269 unknown

GLEAN_10933 H114 SPH 257 unknown

GLEAN_10934 H115 SPH 263 unknown

GLEAN_10935 H116 SPH 257 CG10477 unknown

GLEAN_10936 H117 SPH 266 unknown

GLEAN_10937 H118 SPH 258 unknown

GLEAN_10938 H119 SPH 261 unknown

GLEAN_10939 H120 SPH 255 unknown

GLEAN_10940 P121 SP 262 unknown

GLEAN_10941 H122 SPH 258 unknown

GLEAN_10959 P123 SP 263 unknown

GLEAN_11014 P124 SP 256 CG32271 10

GLEAN_11067 H125 cSPH 241 CG5390 10

GLEAN_11078 P126 cSP 503 Sb 10

GLEAN_11824 P127 SP 277 yip7 9

GLEAN_11825 P128 SP 277 CG10472 9

GLEAN_12390 H129 SPH 722 CG1632 9

GLEAN_12573 H130 SPH 265 CG10477 9

GLEAN_12574 H131 SPH 258 9

GLEAN_12575 H132 SPH 275 9

GLEAN_13042 P133 SP 427 CG10663 5

GLEAN_13084 P134 SP 266 5

GLEAN_13276 P135 SP 295 CG16705 5

GLEAN_13277 P136 cSP 384 easter 5

GLEAN_13278 H137 cSPH 336 easter 5

GLEAN_13279 P138 cSP 359 easter 5

GLEAN_13280 P139 SP 303 easter 5

GLEAN_13326 P140 cSP 349 CG1299 5

GLEAN_13415 P141 SP 287 snake 5

GLEAN_13416 P142 cSP 351 snake 5

GLEAN_13421 H143 SPH 254 5

GLEAN_13613 P144 SP 421 CG7432 5

GLEAN_13709 P145 SP 259 5

GLEAN_13894 H146 SPH 2132 corin 5

GLEAN_14083 P147 SP 258 CG5255 5

GLEAN_14375 H148 SPH 266 5

GLEAN_14391 P149 SP 268 5

GLEAN_14930 P150 SP 261 6

GLEAN_15083 P151 SP 280 CG11911 6

GLEAN_15099 H152 SPH 285 6

GLEAN_15110 P153 SP 792 tequila 6

GLEAN_15130 P154 SP 258 6

GLEAN_15237 P155 SP 281 CG16996 6

GLEAN_15295 P156 SP 446 gd 6

GLEAN_15297 P157 SP 451 CG33329 6

GLEAN_15344 H158 SPH 263 6

GLEAN_15390 H159 SPH 178 6

GLEAN_15579 P160 SP 266 yip7 6

GLEAN_15580 P161 SP 257 6

GLEAN_15617 P162 SP 248 6

GLEAN_15618 P163 SP 247 6

GLEAN_15670 H164 cSPH 691 scarface 6

GLEAN_15779 P165 SP 260 yip7 6

GLEAN_15780 P166 SP 274 jon65aiv 6

GLEAN_16121 P167 SP 245 unknown

GLEAN_16372 P168 SP 264 yip7 unknown

GLEAN_00760-1 serpin1 serpin 355 spn4-PG 2

GLEAN_00760-2 serpin1 serpin 360 spn4-PG 2

GLEAN_02085 serpin2 serpin 758 CG14470 unknown

GLEAN_02247 serpin3 serpin 408 spn4 unknown

GLEAN_04161A serpin4 serpin 445 spn27A unknown

GLEAN_04161B serpin5 serpin 446 spn6 unknown

GLEAN_05065 serpin6 serpin 568 unknown

GLEAN_05740 serpin7 serpin 395 spn4 8

GLEAN_05741A serpin8 serpin 384 8

GLEAN_05741B serpin9 serpin 232 8

GLEAN_05742 serpin10 serpin 258 8

GLEAN_05743 serpin11 serpin 316 8

GLEAN_05744 serpin12 serpin 385 spn2 8

GLEAN_05745A serpin13 serpin 217 8

GLEAN_05745B serpin14 serpin 381 8

GLEAN_05746-1 serpin15 serpin 354 spn2 8

GLEAN_05746-2 serpin15 serpin 355 spn2 8

GLEAN_05747 serpin16 serpin 382 spn7 8

GLEAN_05749 serpin17 serpin 247 8

GLEAN_05750 serpin18 serpin 395 8

GLEAN_05751 serpin19 serpin 396 8

GLEAN_05752-1 serpin20 serpin 394 spn2 8

GLEAN_05752-2 serpin20 serpin 391 spn2 8

GLEAN_05753 serpin21 serpin 456 spn4-PJ 8

GLEAN_05754 serpin22 serpin 402 spn4-PG 8

GLEAN_05771 serpin23 serpin 385 8

GLEAN_06255 serpin24 serpin 390 spn4-PJ 8

GLEAN_06607 serpin25 serpin 386 spn4-PI 8

GLEAN_07869 serpin26 serpin 559 CG7219 4

GLEAN_11718 serpin27 serpin 456 9

GLEAN_13310-1 serpin28 serpin 416 spn5 5

GLEAN_13310-2 serpin28 serpin 413 spn5 5

GLEAN_13389 serpin29 serpin 445 spn2 5

GLEAN_14237 serpin30 serpin 402 CG6680 5

GLEAN_15224 serpin31 serpin 382 spn3 6

GLEAN_00520 spz1 spätzle 227 spz 2

GLEAN_01053 spz7 spätzle 183 2

GLEAN_01054 spz2 spätzle 282 2

GLEAN_05940 spz3 spätzle 1250 spz3 8

GLEAN_06726 spz4 spätzle 334 CG14928 8

GLEAN_13304 spz5 spätzle 342 CG9972 5

GLEAN_16368 spz6 spätzle 411 CG9196 unknown

GLEAN_00625 Toll9 Toll-like receptor 1360 toll-9 (CG7896) 2

GLEAN_00176 Toll1 Toll-like receptor 597 toll 2

GLEAN_04438 Toll3 Toll-like receptor 1046 toll 1=x

GLEAN_04439 Toll4 Toll-like receptor 873 toll 1=x

GLEAN_04452 Toll2 Toll-like receptor 903 toll 1=x

GLEAN_04474 Toll7 Toll-like receptor 1310 toll-7 and 18w 1=x

GLEAN_04895 Toll6 Toll-like receptor 1272 toll-6 1=x

GLEAN_04898 Toll8 Toll-like receptor 1212 tollo 1=x

GLEAN_04901 Toll10 Toll-like receptor 1321 1=x

GLEAN_08202 ML1 MD2-like 144 4

GLEAN_08203 ML2 MD2-like 97 4

GLEAN_14068 ML3 MD2-like 152 5

GLEAN_14069 ML4 MD2-like 150 5

GLEAN_16351 ML5 MD2-like 160 CG3153 unknown

GLEAN_07252 ML6 MD2-like 157 4

GLEAN_14067 ML7 MD2-like 278 5

GLEAN_16352 ML8 MD2-like 156 unknown

GLEAN_02003 cactus cactus 363 cactus unknown

GLEAN_15365 pelle pelle 443 pelle 6

GLEAN_03185 Myd88 Myd88 400 Myd88 3

GLEAN_11895 Tube Tube 643 tube 9

GLEAN_09672 pellino pellino 466 pellino 7

GLEAN_07706 Traf2 Traf 398 traf2 4

GLEAN_08782 cactin cactin 1063 cactin 7

GLEAN_07697 Dif1 REL 556 dif 4

GLEAN_08096 Dif2 REL 384 dlf 4

GLEAN_14042 FADD FADD 195 fadd 5

GLEAN_01419 IKKb IKKb 704 ird5 unknown

GLEAN_09798 IKKb IKKb 732 ik2 7

GLEAN_00541 IKKg IKKg 503 2

GLEAN_10851 IMD IMD 199 imd unknown

GLEAN_05572 TAK1 TAK 511 tak1 8

GLEAN_03841 casps1 caspase 309 3

GLEAN_12581 casps2 caspase 389 9

GLEAN_12580 casps3 caspase 395 9

GLEAN_14026 casps4 dredd/casp8 568 dredd 5

GLEAN_00105 casps5 caspase 317 unknown

GLEAN_12579 casps6 caspase 402 nc 9

GLEAN_02397 casps7 caspase 545 nc 3

GLEAN_00068 casps8 caspase 368 unknown

GLEAN_09985 caspar blocking caspase 656 CG8400 7

GLEAN_01189 IAP2 IAP 494 iap2 2

GLEAN_01192 IAP1 IAP 333 2

GLEAN_09848 IAP3 IAP 4336 bruce 7

GLEAN_02709 IAP4 IAP 142 3

GLEAN_11191 REL1 REL 853 relish 10

GLEAN_14708 REL2 REL 1048 NFAT 5

GLEAN_05952 Tab2 Tab2 527 tab2 8

GLEAN_00385 Hep Hep 645 hep 2

GLEAN_06810 basket1 basket 390 basket 8

GLEAN_11967 basket2 basket 372 p38b 9

GLEAN_13594 basket3 basket 352 p38b 5

GLEAN_06814 Jra Jra 390 jra 8

GLEAN_11870 kay kay 366 kay 9

GLEAN_01874 DOME DOME 1100 dome unknown

GLEAN_08648 HOP HOP 503 hop 7

GLEAN_13218 STAT STAT 780 stat92E 5

GLEAN_00325 proPO1 prophenoloxidase 682 CG8193 2

GLEAN_14907 proPO2 prophenoloxidase 683 CG8193 6

GLEAN_15848 proPO3 prophenoloxidase 683 CG8193 6

GLEAN_06342 MI melanization inhibitor 354 1

GLEAN_05374 hexamerin1 hexamerin 695 lsp2 8

GLEAN_05375 hexamerin2 hexamerin 701 lsp2 8

GLEAN_05376 hexamerin3 hexamerin 748 lsp2 8

GLEAN_05377 hexamerin4 hexamerin 769 lsp2 8

GLEAN_06515 hexamerin5 hexamerin 703 lsp2 8

GLEAN_06769 hexamerin6 hexamerin 698 lsp2 8

GLEAN_11385 catalase1 catalase 516 cat 10

GLEAN_11090 catalase2 catalase 479 cat 10

GLEAN_00083 catalase3 catalase 229 cat unknown

GLEAN_00084 catalase4 catalase 221 cat unknown

GLEAN_10362 GTX1 glutathione oxidase 199 PHGPx 1

GLEAN_10355 GTX2 glutathione oxidase 247 PHGPx 1

GLEAN_10354 GTX3 glutathione oxidase 198 PHGPx 1

GLEAN_05493 HPX1 heme peroxidase 1466 CG10211 8

GLEAN_15234 HPX2 heme peroxidase 1076 CG6879 6

GLEAN_11222 HPX3 heme peroxidase 903 CG5873 10

GLEAN_04579 HPX4 heme peroxidase 772 CG6969 1

GLEAN_04551 HPX5 heme peroxidase 741 pxd 1

GLEAN_00751 HPX6 heme peroxidase 727 pxt 2

GLEAN_00175 HPX7 heme peroxidase 676 pxd 2

GLEAN_04661 HPX8 heme peroxidase 603 pxd 1

GLEAN_01556 HPX9 heme peroxidase 1388 pxn unknown

GLEAN_02498 HPX10 heme peroxidase 1515 CG3131 3

GLEAN_04592 HPX11 heme peroxidase 951 CG3131 1

GLEAN_14929 TPX6 peroxiredoxin 196 Jafrac1 (CG1633) 2

GLEAN_01700 TPX2 peroxiredoxin 196 Jafrac1 (CG1633) 9

GLEAN_12328 TPX1 peroxiredoxin 233 prx5037 (CG5826) 2

GLEAN_01071 TPX3 peroxiredoxin 243 Jafrac2 (CG1274) 5

GLEAN_13791 TPX5 peroxiredoxin 219 prx6005 (CG3083) 1

GLEAN_04948 TPX4 peroxiredoxin 223 prx2540 1

GLEAN_07011 SOD3 superoxide dismutase 153 sod (CG11793) 4

GLEAN_11676 SOD2 superoxide dismutase 166 CG9027 9

GLEAN_11675 SOD4 superoxide dismutase 227 ccs 9

GLEAN_11770 SOD1 superoxide dismutase 1111 CG31028 9

GLEAN_07737 attacin1 antimicrobial peptide 165 4

GLEAN_07738 attacin2 antimicrobial peptide 145 4

GLEAN_07739 attacin3 antimicrobial peptide 148 4

GLEAN_00499 cecropin1 antimicrobial peptide pseudogene 2

Cec2 cecropin2 antimicrobial peptide 93 2

GLEAN_00500 cecropin3 antimicrobial peptide 90 2

GLEAN_06250 defensin1 antimicrobial peptide 79 8

GLEAN_10517 defensin2 antimicrobial peptide 79 unknown

GLEAN_12469 defensin3 antimicrobial peptide 83 9

Def4 defensin4 antimicrobial peptide 82

GLEAN_05093 coleoptericin1 antimicrobial peptide 141 unknown

GLEAN_05096 coleoptericin2 antimicrobial peptide 141 unknown

GLEAN_10349 lysozyme1 lysozyme 275 unknown

GLEAN_10350 lysozyme2 lysozyme 147 unknown

GLEAN_10351 lysozyme3 lysozyme 148 unknown

GLEAN_10352 lysozyme4 lysozyme 142 unknown

GLEAN_11324 WAP antimicrobial peptide? 122 10

GLEAN_01889 neuroglian neuroglian/hemolin 1267 nrg unknown

GLEAN_00948 SR-B6 scavenger receptor 529 CG10345 2

GLEAN_07247 SR-B7 scavenger receptor 463 crq 4

GLEAN_07861 SR-A1 scavenger receptor 2993 CG3921 4

GLEAN_08191 SR-B10 scavenger receptor 518 CG7000 4

GLEAN_08209 SR-B1 scavenger receptor 507 CG7000 4

GLEAN_08210 SR-B3 scavenger receptor 1410 CG7000 4

GLEAN_10348 SR-B11 scavenger receptor 408 CG7000 unknown

GLEAN_10353 SR-B12 scavenger receptor 507 CG7000 unknown

GLEAN_10356 SR-B13 scavenger receptor 1240 CG7000 unknown

GLEAN_11653 SR-A2 scavenger receptor 481 lox2 9

GLEAN_12756 SR-B14 scavenger receptor 496 CG12789 9

GLEAN_12757 SR-B15 scavenger receptor 377 CG12789 9

GLEAN_12758 SR-B16 scavenger receptor 540 CG12789 9

GLEAN_13894 SR-A3 scavenger receptor 2132 corin 5

GLEAN_14946 SR-B5 scavenger receptor 569 6

GLEAN_14951 SR-B8 scavenger receptor 550 CG3829 6

GLEAN_14954 SR-B9 scavenger receptor 515 emp 6

GLEAN_15110 SR-A4 scavenger receptor 792 tequila 6

GLEAN_15144 SR-B4 scavenger receptor 547 6

GLEAN_15640 SR-C scavenger receptor 511 sr-CII 6

GLEAN_15854 SR-B2 scavenger receptor 557 CG1887 6

GLEAN_11427 NimA nimrod 445 10

GLEAN_11428 NimB nimrod 355 10

GLEAN_02053 NimCl1 nimrod 847 unknown

GLEAN_15258 NimCl2 nimrod 436 6

GLEAN_00689 draper draper 993 draper 2

* Seventy-five SPs or SPHs which are shorter than 280 residues are not expected to function in physiological processes requiring

sophisticated protein-protein interactions (*e.g.*, immune responses).
