# Supplementary figures and images for: Comparative genomic analysis of the Tribolium immune system
Source: Genome Biol. 2007 Aug 29;8(8):R177. doi: 10.1186/gb-2007-8-8-r177 (PMC2375007; doi:10.1186/gb-2007-8-8-r177)

## Slide 1
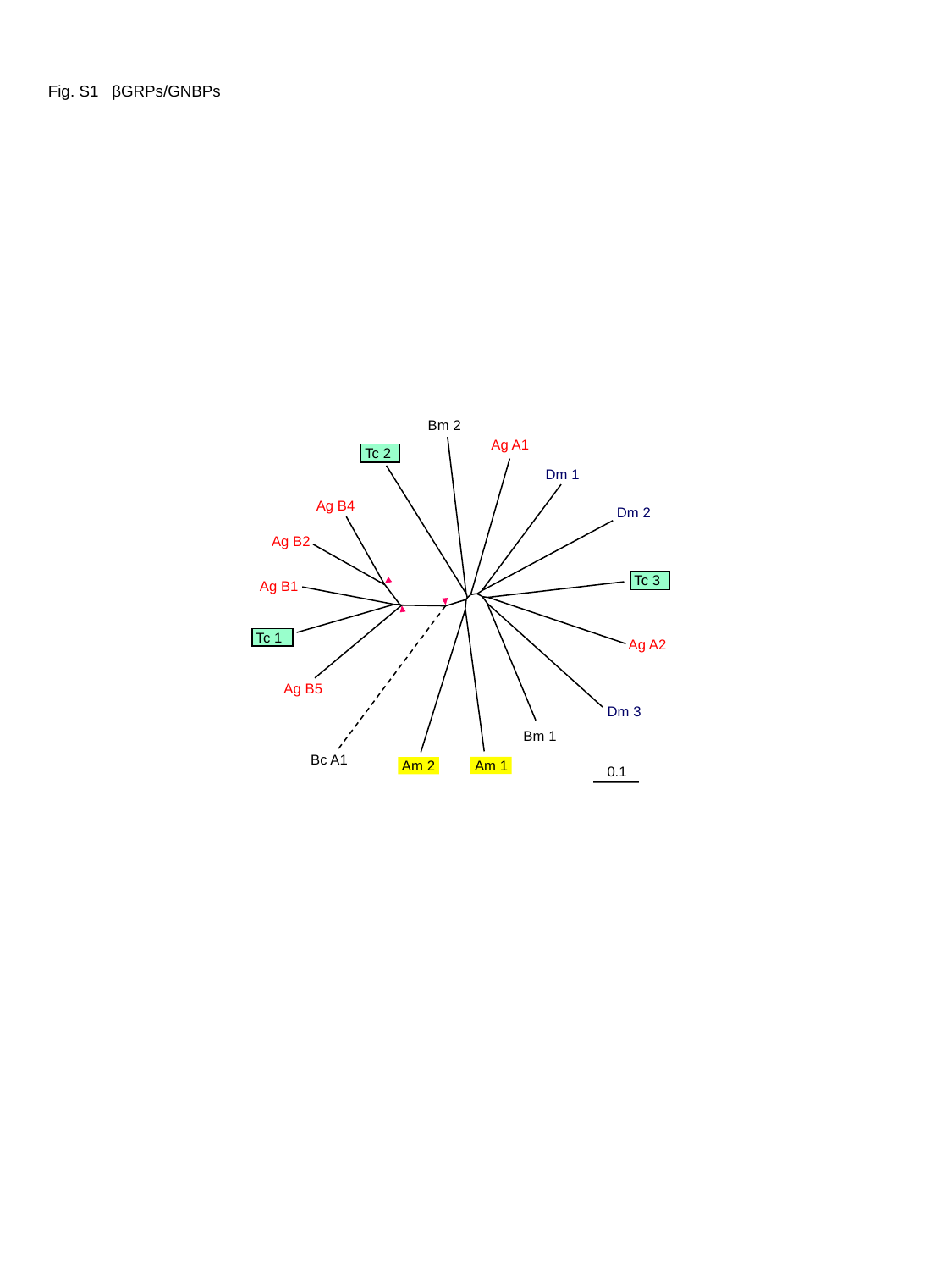

Fig. S1 βGRPs/GNBPs
Bm 2
Ag A1
0.1
 Tc 2
Dm 1
Ag B4
Dm 2
Ag B2
 Tc 3
Ag B1
 Tc 1
Ag A2
Ag B5
Dm 3
Bm 1
Bc A1
 Am 1
 Am 2

Supplement: Additional data file 2 — The sequences of three Tribolium (Tc), three Drosophila (Dm), two Apis (Am), six Anopheles (Ag) and two Bombyx βGRPs/GNBPs are aligned with Bacillus circulans (Bc) β-1,3-glucanase A1 as an outgroup. There was a family expansion in the lineage of A. gambiae. Pink arrowheads indicate nodes with bootstrap values greater than 800 from 1,000 trials, and the dashed line marks the outgroup. [file gb-2007-8-8-r177-S2.ppt]

## Slide 1
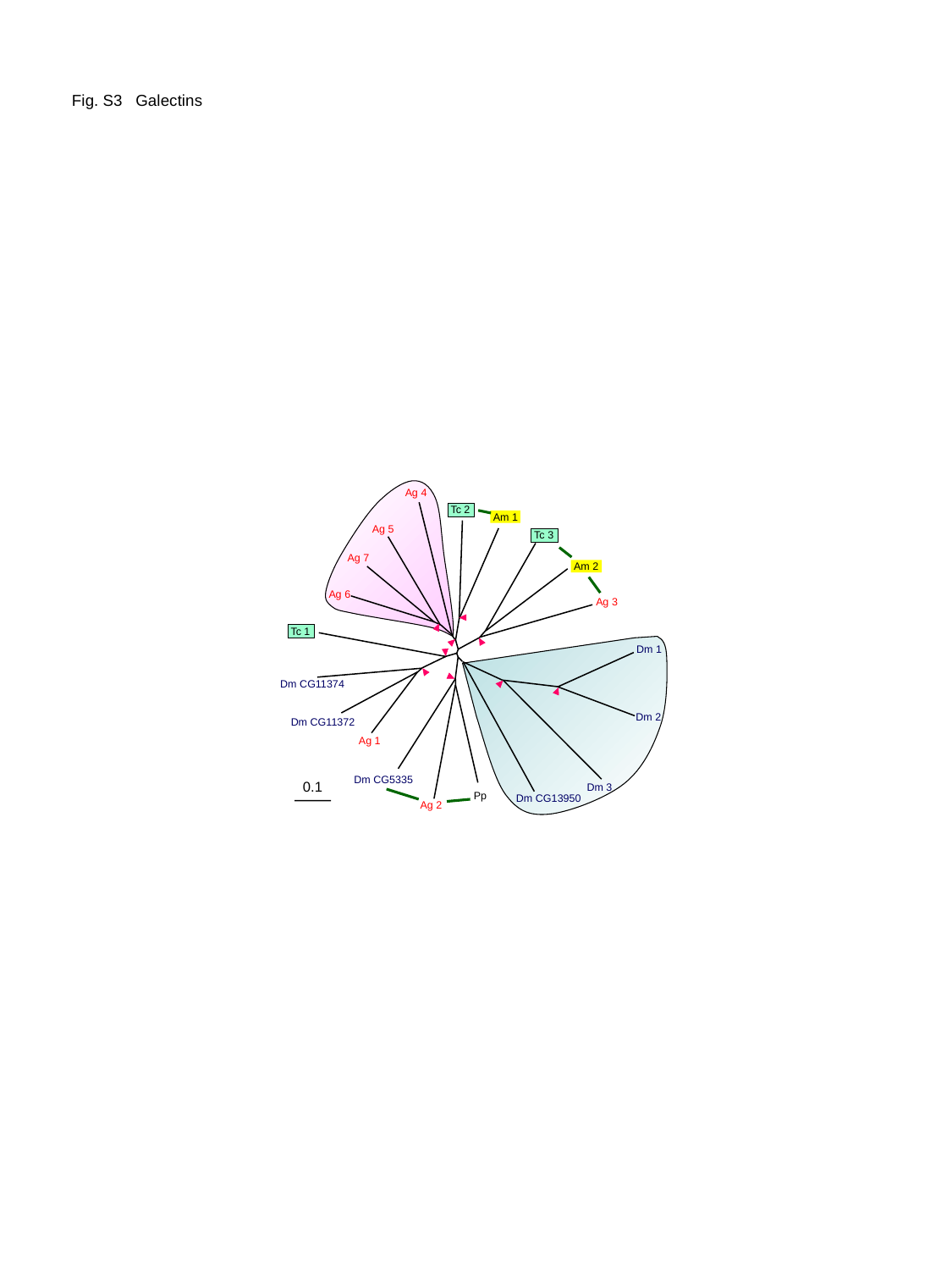

Fig. S3 Galectins
Ag 4
 Tc 2
 Am 1
Ag 5
 Tc 3
Ag 7
 Am 2
Ag 6
Ag 3
 Tc 1
Dm 1
Dm CG11374
Dm 2
Dm CG11372
Ag 1
Dm CG5335
0.1
Dm 3
Pp
Dm CG13950
Ag 2

Supplement: Additional data file 4 — The amino acid sequences from three Tribolium (Tc), seven Drosophila (Dm), seven Anopheles (Ag), two Apis (Am) and one Phlebotomus (Pp) galectins are examined. The phylogenetic tree, derived from the aligned sequences, shows family expansions in Anopheles (pink) and Drosophila (blue). Pink arrowheads at nodes denote bootstrap values greater than 800 from 1,000 trials. Green lines connect the putative orthologous pairs or trio. [file gb-2007-8-8-r177-S4.ppt]

## Slide 1
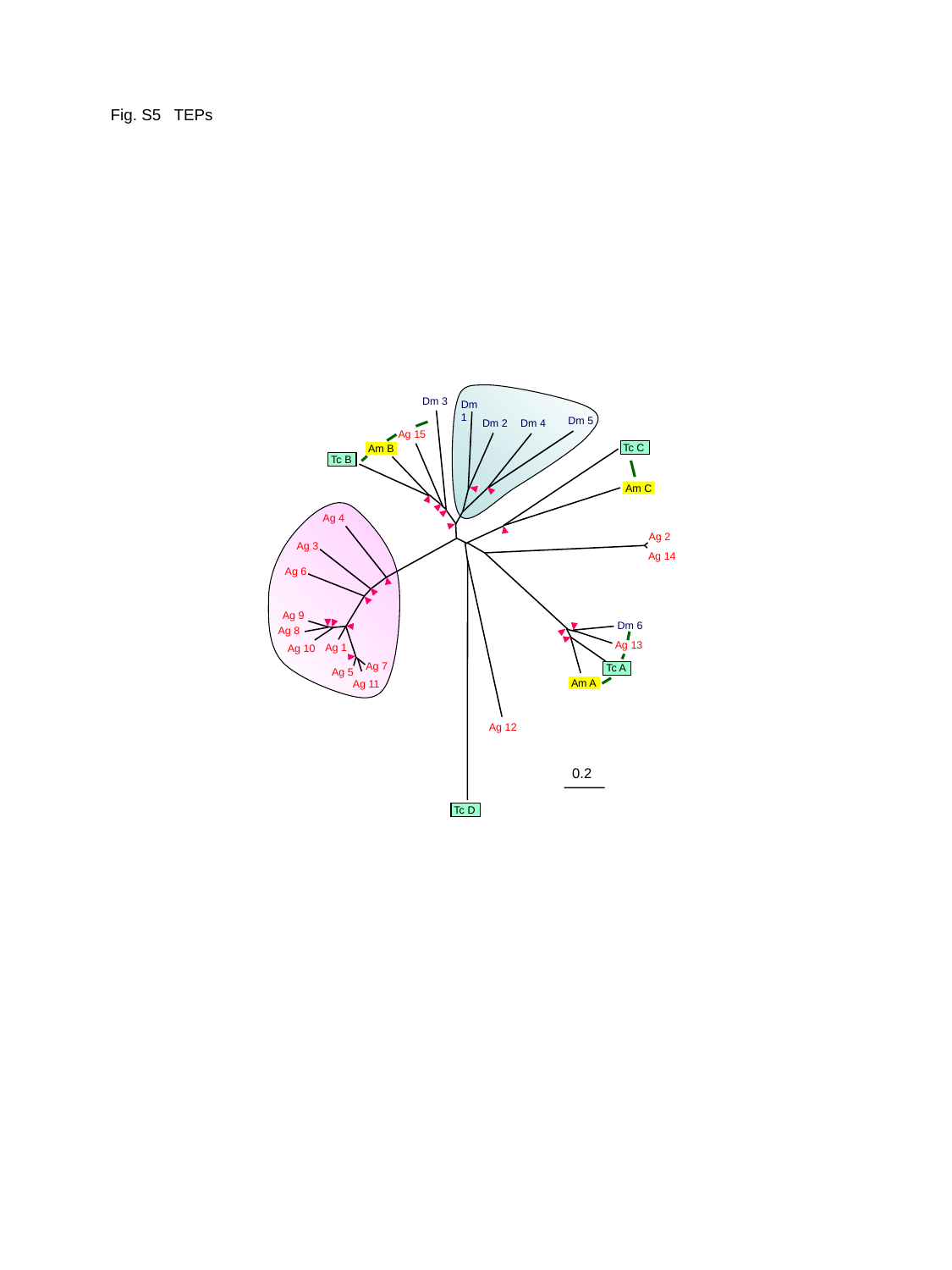

Fig. S5 TEPs
Dm 3
Dm 1
Dm 5
Dm 2
Dm 4
Ag 15
 Tc C
 Am B
 Tc B
 Am C
Ag 4
Ag 2
Ag 3
Ag 14
Ag 6
Ag 9
Dm 6
Ag 8
Ag 13
Ag 1
Ag 10
Ag 7
 Tc A
Ag 5
 Am A
Ag 11
Ag 12
0.2
 Tc D

Supplement: Additional data file 6 — The sequences of four Tribolium (Tc), six Drosophila (Dm), fifteen Anopheles (Ag) and three Apis (Am) TEPs are aligned. Lineage-specific family expansions are indicated with color shades (blue for Drosophila and pink for Anopheles). Pink arrowheads at nodes denote bootstrap values greater than 800 for 1,000 trials, and green bars link the predicted 1:1 and 1:1:1:1 orthologs. [file gb-2007-8-8-r177-S6.ppt]

## Slide 1
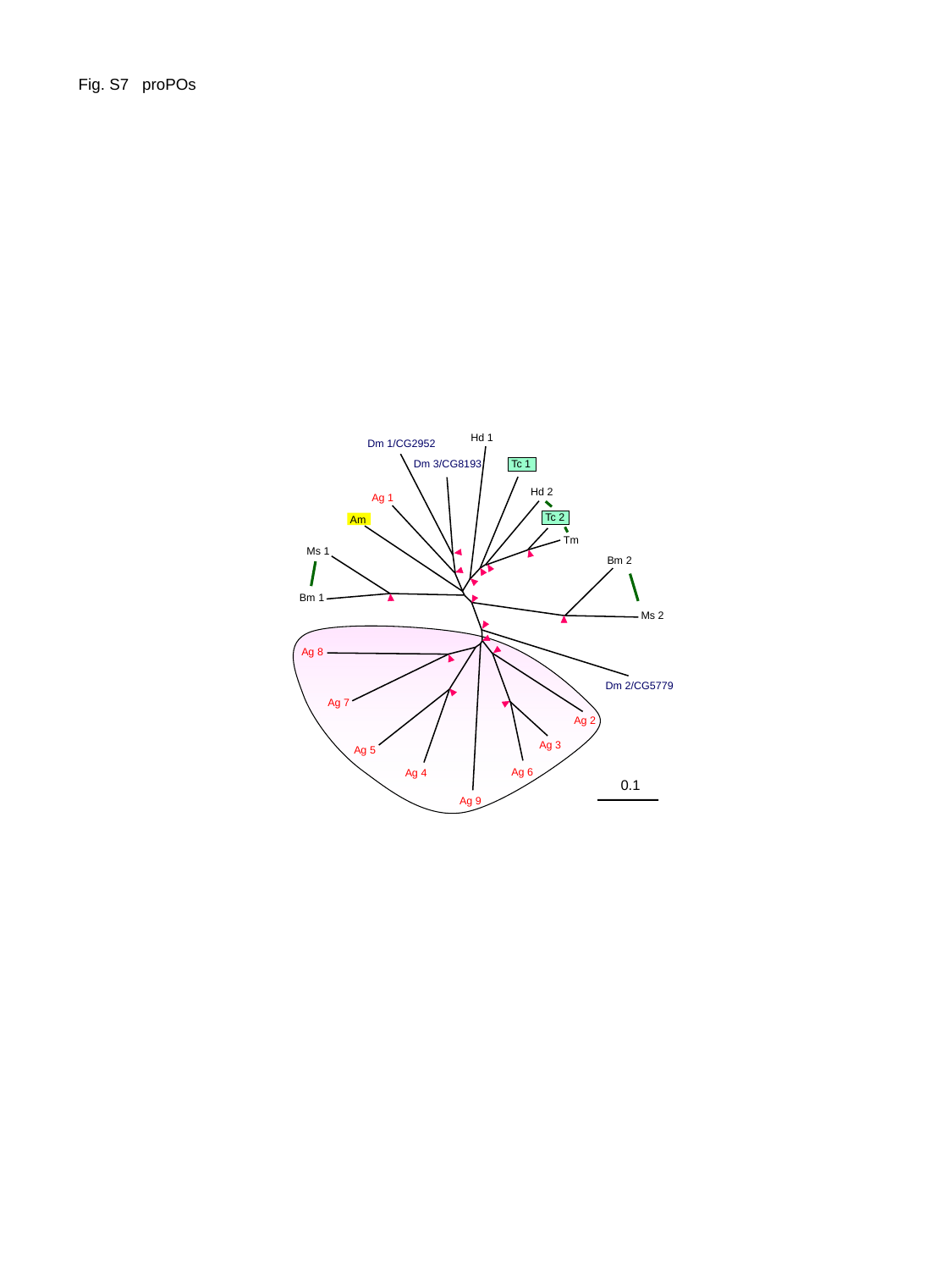

Fig. S7 proPOs
Hd 1
Dm 1/CG2952
Dm 3/CG8193
 Tc 1
Hd 2
Ag 1
 Tc 2
 Am
Tm
Ms 1
Bm 2
Bm 1
Ms 2
Ag 8
Dm 2/CG5779
Ag 7
Ag 2
Ag 3
Ag 5
Ag 6
Ag 4
0.1
Ag 9

Supplement: Additional data file 8 — The entire sequences of Tribolium (Tc), Tenebrio (Tm), Holotrichia (Hd), and Drosophila (Dm), Anopheles (Ag), Apis (Am), Bombyx (Bm) and Manduca (Ms) proPOs are compared. Tribolium proPO3, >99% identical in amino acid sequence to Tc-proPO2, is not included in the analysis. The phylogenetic tree, derived from the multiple sequence alignment, shows the extensive family expansion (shaded pink) in the malaria mosquito. Pink arrowheads point to nodes with high bootstrap values (>800 from 1,000 trials), and green lines link the predicted 1:1 or 1:1:1 orthologs. [file gb-2007-8-8-r177-S8.ppt]
