# Supplementary material for: Comparative genomic analysis of the Tribolium immune system
Source: Genome Biol. 2007 Aug 29;8(8):R177. doi: 10.1186/gb-2007-8-8-r177 (PMC2375007; doi:10.1186/gb-2007-8-8-r177)
Supplement: Additional data file 3 — The sequences of sixteen Tribolium (Tc), ten Drosophila (Dm), eight Anopheles (Ag) and eight Apis (Am) sequences are aligned. TcCTL3 (that is, Tc 3) contains two carbohydrate recognition domains and the first one is used for comparison. Different CTL subfamilies (GA, galactose; MA, mannose) are indicated, with the predicted orthologous groups marked by blue dots (for 1:1, 1:1:1 and 1:1:1:1 relationships). Pink arrowheads indicate nodes with significant bootstrap values (>800 of 1,000 trials). Note that many Dm- and Ag-CTLs, not included in this analysis, are results of major lineage-specific expansions [29]. [file gb-2007-8-8-r177-S3.ppt]

## Slide 1
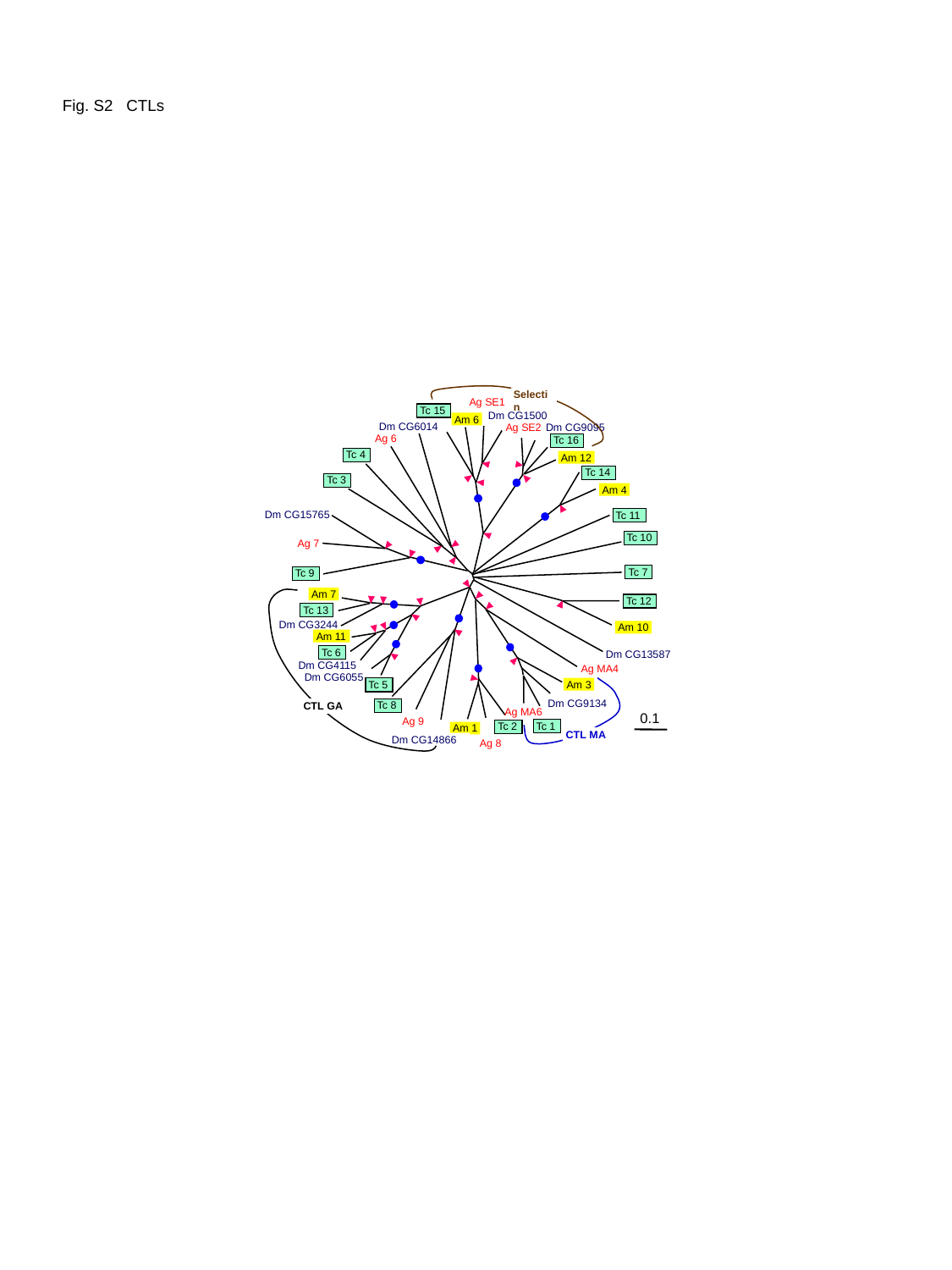

Fig. S2 CTLs
Selectin
Ag SE1
 Tc 15
Dm CG1500
 Am 6
Dm CG6014
Ag SE2
Dm CG9095
Ag 6
 Tc 16
 Tc 4
 Am 12
 Tc 14
 Tc 3
 Am 4
Dm CG15765
 Tc 11
 Tc 10
Ag 7
 Tc 7
 Tc 9
 Am 7
 Tc 12
 Tc 13
Dm CG3244
 Am 10
 Am 11
 Tc 6
Dm CG13587
Dm CG4115
Ag MA4
Dm CG6055
 Am 3
 Tc 5
Dm CG9134
 Tc 8
CTL GA
Ag MA6
0.1
Ag 9
 Tc 1
 Tc 2
 Am 1
CTL MA
Dm CG14866
Ag 8
