# Supplementary material for: Comparative genomic analysis of the Tribolium immune system
Source: Genome Biol. 2007 Aug 29;8(8):R177. doi: 10.1186/gb-2007-8-8-r177 (PMC2375007; doi:10.1186/gb-2007-8-8-r177)
Supplement: Additional data file 5 — The sequences of seven Tribolium (Tc), fourteen Drosophila (Dm), nine Anopheles (Ag), nine Aedes (Aa) and one Apis (Am) FREPs are aligned for constructing this unrooted tree. For simplicity, other family members from Drosophila, Anopheles and Aedes are excluded from the analysis. Lineage-specific expansions (shaded yellow for Tribolium, blue for Drosophila and pink for Anopheles) are confirmed in the complete tree that includes all FREPs from these four species (data not shown). Nodes with pink arrowheads have bootstrap values exceeding 800 in 1,000 trials. Green bars connect the putative orthologs with 1:1 or 1:1:1 relationship. The chromosomal locations (lower corner) of Tribolium FREP-1 through -4 are shown. [file gb-2007-8-8-r177-S5.ppt]

## Slide 1
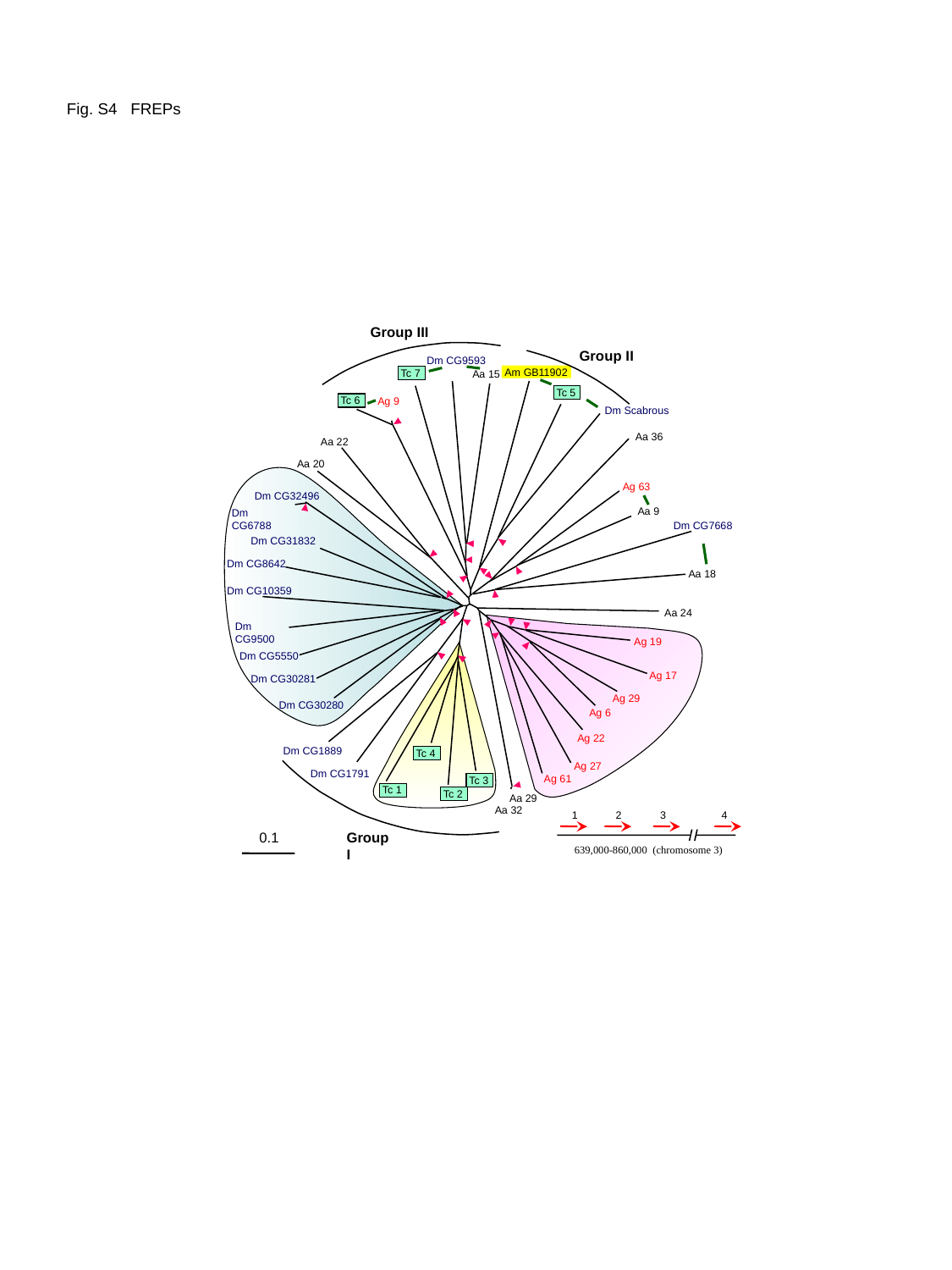

Fig. S4 FREPs
Group III
Group II
Dm CG9593
 Am GB11902
 Tc 7
Aa 15
 Tc 5
 Tc 6
Ag 9
Dm Scabrous
Aa 36
Aa 22
Aa 20
Ag 63
Dm CG32496
Aa 9
Dm CG6788
Dm CG7668
Dm CG31832
Dm CG8642
Aa 18
Dm CG10359
Aa 24
Dm CG9500
Ag 19
Dm CG5550
Ag 17
Dm CG30281
Ag 29
Dm CG30280
Ag 6
Ag 22
Dm CG1889
 Tc 4
Ag 27
Dm CG1791
Ag 61
 Tc 3
 Tc 1
 Tc 2
Aa 29
Aa 32
 1
 2
 3
 4
 639,000-860,000 (chromosome 3)
Group I
0.1
