# Supplementary material for: Comparative genomic analysis of the Tribolium immune system
Source: Genome Biol. 2007 Aug 29;8(8):R177. doi: 10.1186/gb-2007-8-8-r177 (PMC2375007; doi:10.1186/gb-2007-8-8-r177)
Supplement: Additional data file 7 — The amino acid sequences of seven Tribolium (Tc), six Drosophila (Dm), five Anopheles (Ag), two Apis (Am) and one Bombyx (Bm) spätzles are aligned for building the unrooted tree. Pink arrowheads indicate nodes with significant bootstrap values (>800 of 1,000 trials), and green bars connect the putative orthologous pairs or trios. [file gb-2007-8-8-r177-S7.ppt]

## Slide 1
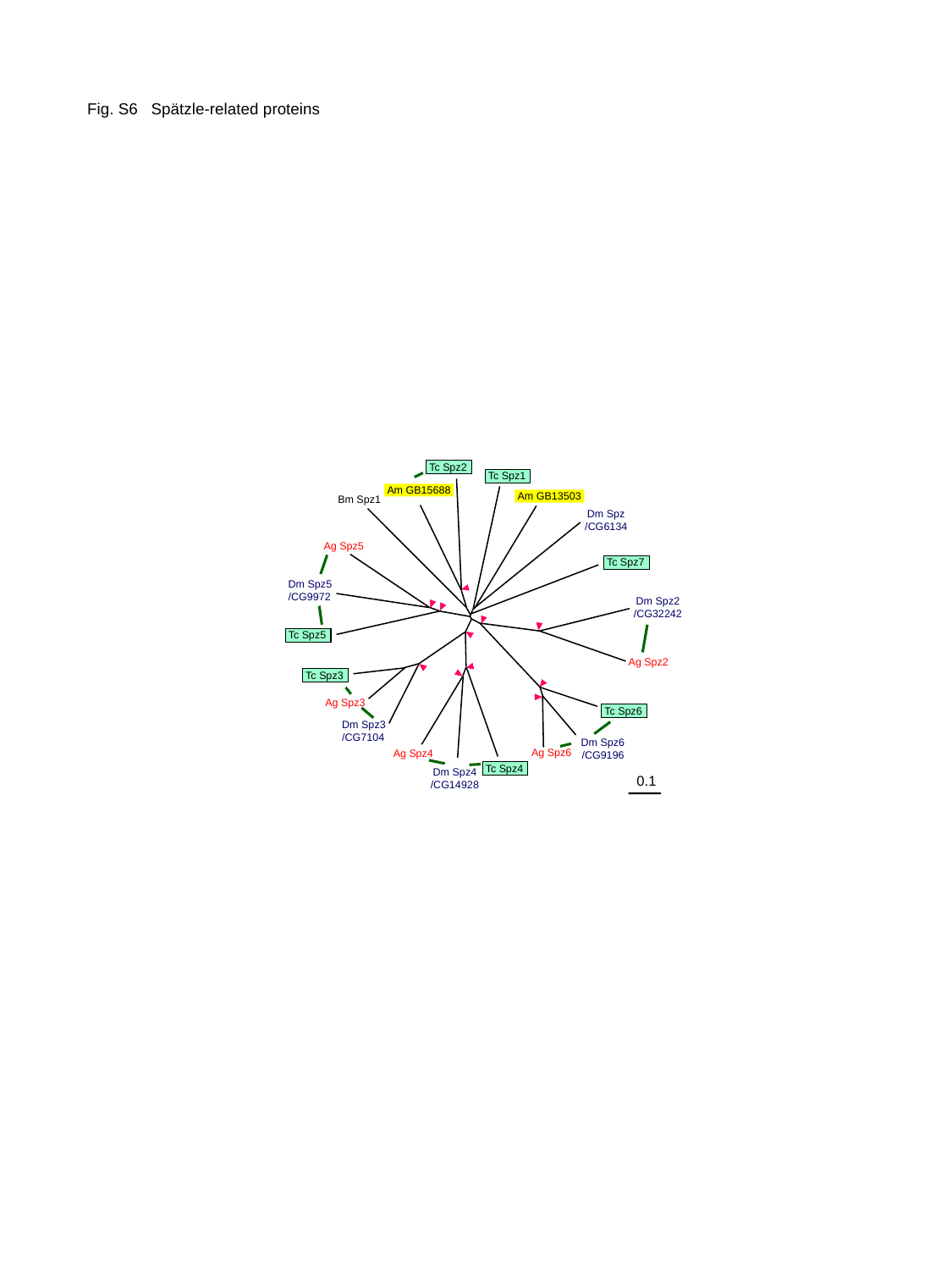

Fig. S6 Spätzle-related proteins
 Tc Spz2
 Tc Spz1
 Am GB15688
 Am GB13503
Bm Spz1
Dm Spz
/CG6134
Ag Spz5
 Tc Spz7
Dm Spz5
/CG9972
Dm Spz2
/CG32242
 Tc Spz5
Ag Spz2
 Tc Spz3
Ag Spz3
 Tc Spz6
Dm Spz3
/CG7104
Dm Spz6
/CG9196
Ag Spz6
Ag Spz4
 Tc Spz4
Dm Spz4
/CG14928
0.1
