# Supplementary material for: Comparative genomic analysis of the Tribolium immune system
Source: Genome Biol. 2007 Aug 29;8(8):R177. doi: 10.1186/gb-2007-8-8-r177 (PMC2375007; doi:10.1186/gb-2007-8-8-r177)
Supplement: Additional data file 9 — (a) GTX, (b) SOD and (c) TPX. The Tribolium (Tc), Drosophila (Dm), Anopheles (Ag) and Apis sequences are studied. As shown in the trees, duplication and divergence have given rise to gene clusters (shaded yellow for Tribolium and blue for Drosophila). Pink arrowheads denote nodes with high bootstrap values (>800 in 1,000 trials), whereas green lines connect the putative orthologs with 1:1, 1:1:1 or 1:1:1:1 relationships. [file gb-2007-8-8-r177-S9.ppt]

## Slide 1
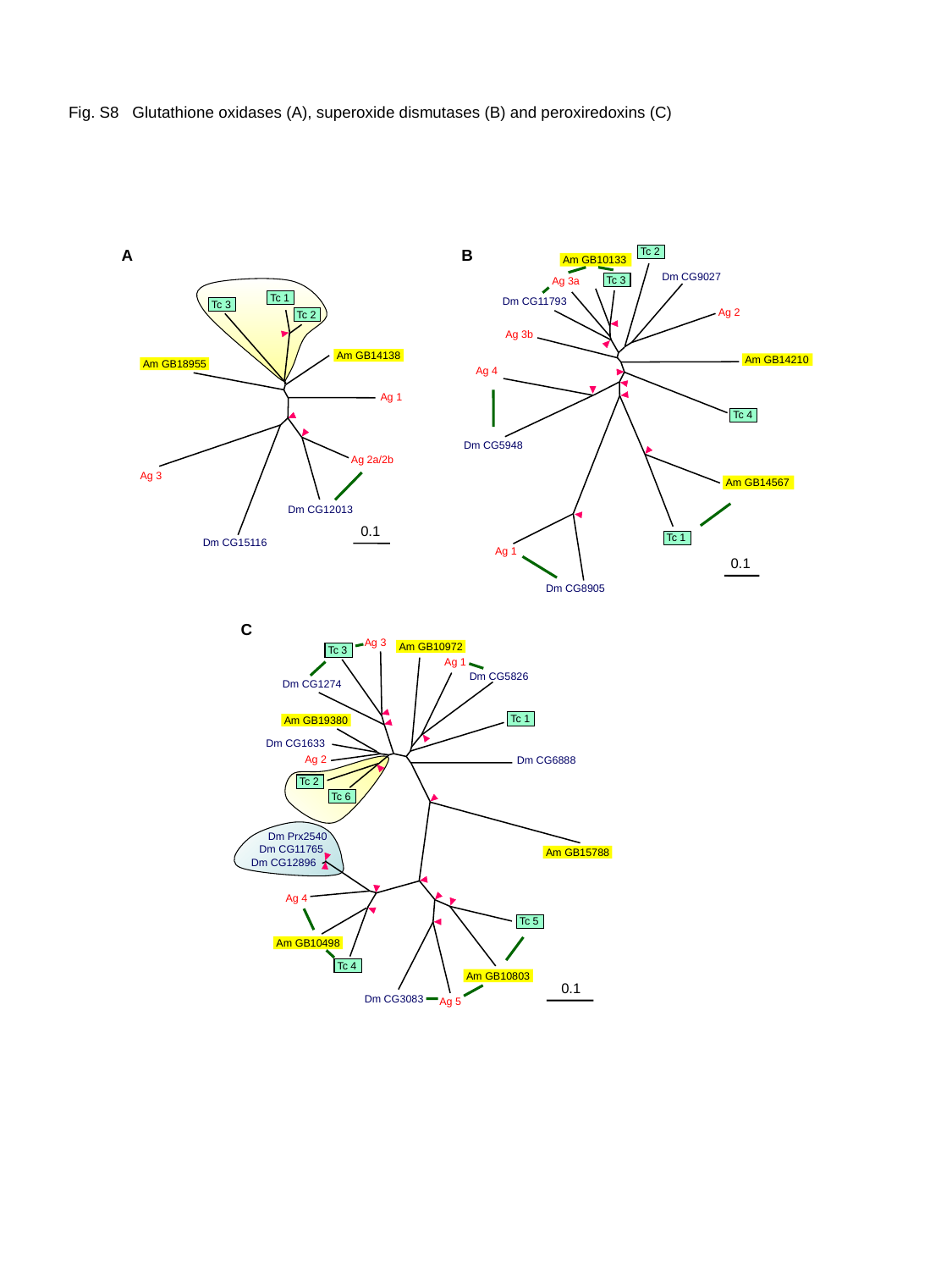

Fig. S8 Glutathione oxidases (A), superoxide dismutases (B) and peroxiredoxins (C)
 Tc 2
 Am GB10133
Dm CG9027
 Tc 3
Ag 3a
Dm CG11793
Ag 2
Ag 3b
 Am GB14210
Ag 4
 Tc 4
Dm CG5948
 Am GB14567
 Tc 1
Ag 1
0.1
Dm CG8905
A
B
 Tc 1
 Tc 3
 Tc 2
 Am GB14138
 Am GB18955
Ag 1
Ag 2a/2b
 Ag 3
Dm CG12013
0.1
Dm CG15116
C
Ag 3
 Am GB10972
 Tc 3
Ag 1
Dm CG5826
Dm CG1274
 Tc 1
 Am GB19380
Dm CG1633
Ag 2
Dm CG6888
 Tc 2
 Tc 6
Dm Prx2540
Dm CG11765
 Am GB15788
Dm CG12896
Ag 4
 Tc 5
 Am GB10498
 Tc 4
 Am GB10803
0.1
Dm CG3083
Ag 5
