# Supplementary material for: Comparative genomic analysis of the Tribolium immune system
Source: Genome Biol. 2007 Aug 29;8(8):R177. doi: 10.1186/gb-2007-8-8-r177 (PMC2375007; doi:10.1186/gb-2007-8-8-r177)
Supplement: Additional data file 10 — The sequences of four Tribolium (Tc), twelve Drosophila (Dm), five Anopheles (Ag), one Bombyx (Bm), one Manduca (Ms), and two Apis (Am) lysozymes are aligned and used to derive the tree (upper panel). Lineage-specific expansion (shaded in different colors) occurs quite extensively in this family of enzymes. For instance, four Tribolium lysozyme genes are found as a gene cluster (lower panel) at the same genomic location. Pink arrowheads at nodes indicate bootstrap values greater than 800 from 1,000 trials. A green bar links the putative orthologous pair. [file gb-2007-8-8-r177-S10.ppt]

## Slide 1
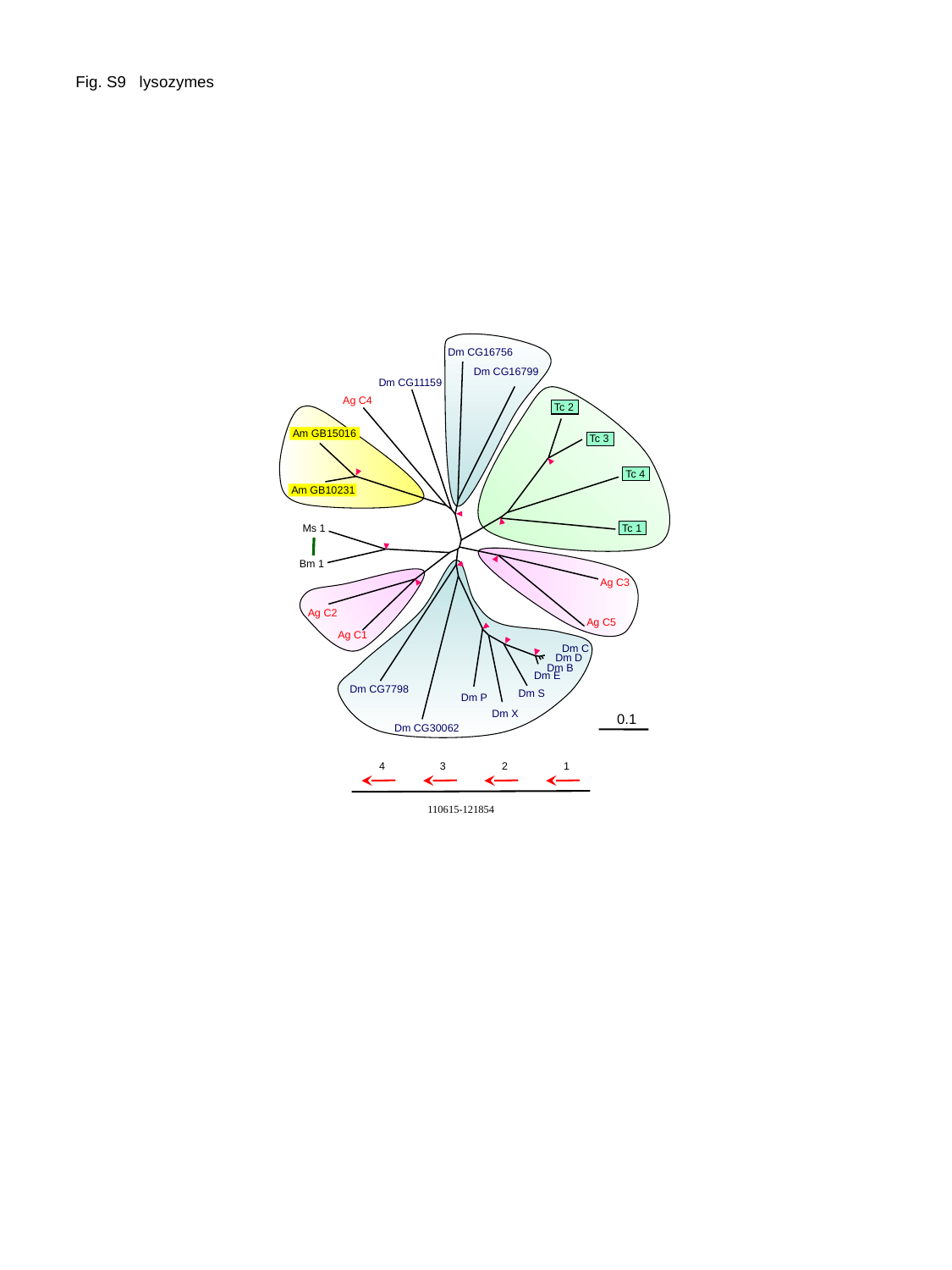

Fig. S9 lysozymes
Dm CG16756
Dm CG16799
Dm CG11159
Ag C4
 Tc 2
 Am GB15016
 Tc 3
 Tc 4
 Am GB10231
 Tc 1
Ms 1
Bm 1
Ag C3
Ag C2
Ag C5
Ag C1
Dm C
Dm D
Dm B
Dm E
Dm CG7798
Dm S
Dm P
Dm X
0.1
Dm CG30062
 4
 3
 2
 1
110615-121854
