# Supplementary material for: Comparative genomic analysis of the Tribolium immune system
Source: Genome Biol. 2007 Aug 29;8(8):R177. doi: 10.1186/gb-2007-8-8-r177 (PMC2375007; doi:10.1186/gb-2007-8-8-r177)
Supplement: Additional data file 11 — Functions, families, and counts of putative defense proteins from D. melanogaster, A. gambiae, A. mellifera and T. castaneum [file gb-2007-8-8-r177-S11.doc]

**Table S2 Functions, families, and counts of putative defense proteins from the insects**

| **Protein family** | ***D. melanogaster**** | ***A. gambiae**** | ***A. mellifera**** | ***T. castaneum*** |
| --- | --- | --- | --- | --- |

*Recognition*

PGRP 13 7 4 7

βGRP/GNBP 3 6 2 3

galectin 5 8 2 3

C-type lectin (CTL) 35 22 10 16

FREP 13 57 2 7

*Signaling*

clip-domain SP/SPH 37 41 18 48

serpin 28 14 5 31

spätzle 6 6 2 7

Toll-like receptor 9 11 5 9

Cactus 1 1 3 1

Rel transcription factor 5 3 4 4

*Execution*

prophenoloxidase 3 9 1 3

defensin 1 4 2 4

other antimicrobial peptide 19 5 4 8

lysozyme 14 6 3 4

TEP 6 15 3 4

SR-B 12 16 10 16

glutathione oxidase 2 3 2 3

peroxiredoxin 8 5 5 6

SOD 4 5 3 4

| ***Total*** | **224** | **244** | **90** | **188** |
| --- | --- | --- | --- | --- |

* *Drosophila*, *Anopheles* and *Apis* counts are based on the previous reports [6, 7] and newer analyses.
