# Supplementary material for: Comparative genomic analysis of the Tribolium immune system
Source: Genome Biol. 2007 Aug 29;8(8):R177. doi: 10.1186/gb-2007-8-8-r177 (PMC2375007; doi:10.1186/gb-2007-8-8-r177)
Supplement: Additional data file 12 — Oligonucleotide primers used in expression analysis by real-time PCR [file gb-2007-8-8-r177-S12.doc]

**Table S3 Oligonucleotide primers used in expression analysis by real-time PCR**

| **Gene ID** | **Gene name** | **Forward primer** (5’ to 3’) | **Reverse primer** (5’ to 3’) | **Length** (bp) |
| --- | --- | --- | --- | --- |

GLEAN_02789 PGRP-LA TGCGACTCCAAGACTTCCTT TCGGGGTAACAGTGTGTGAA 137

GLEAN_10508 PGRP-LE GCCAAGGAGTTGATTCGGTA CACCGTTCCCAACTCTTGAT 137

GLEAN_10611 PGRP-SA TCGTGAGTCGCACTAGATGG CTTCCTCAGTGCTGCAAGTG 117

GLEAN_13620 PGRP-SB GTCTAAGTCTCCAGGCGTGC TTGTAACCGATGTCTTGCCA 88

GLEAN_02295 GNBP1 GCTCGTATTCGCACTGTCAA AGGCCAACCGGAGTAAAGAT 135

GLEAN_03991 GNBP3 CTGTAAAGCAAGCGCAACAA CGGTTCCACAACTCTGGTTT 102

GLEAN_14053 CTL7 GTGTCCGGAAGTCCACAACT TCTGAGACAAGTTGCGTTCG 112

GLEAN_07619 GALE1 GCAAAACAAAACCGAATCGT ACTCCGGTGACATAAATCGC 99

GLEAN_11871 GALE2 AACACCTCTATGCCAATGCC TTATGGTGGTGGTGGTGATG 131

GLEAN_09667 TEP-C TACACAGGACCTATTCCGCC AGAGCCACGTCTCTGGAAAA 118

GLEAN_00247 cSPH2 ACTACATCGACGTGTGCTGC GTTTTCCGTGTTTCCGGTTA 140

GLEAN_05976 cSP66 ACCCATCTGTTGAGAGCCAC TTAAGGTTGTGGGAGCCTTG 131

GLEAN_13277 cSP136 CGATCCGAATGACGTAAACC AGCAGATGGGTCGGACATAG 92

GLEAN_13389 sepin29 TGCAATTGAGGTGGAAATGA CCTATGCCGTCTTTGGGTAA 141

GLEAN_14237 sepin30 GCCACGTTTTACCGTCAAGT CAAATAGTGGGGGAACATGC 118

GLEAN_00520 spätzle1 TGATTGTCAATGTGGAGGGA TAGGAGGTGTACCCTGTCGG 106

GLEAN_00176 Toll1 GATTGGGTACCGGGTGAAAT GGCCCAAACACTTTGAAGAA 108

GLEAN_04452 Toll2 CCGTCGTTTTGGCTGATAAT TCCAGTTCGCGAGCTTTATT 129

GLEAN_04438 Toll3 GGCTTACAAGAGCTGCAACC GATAAAGCGTCTTTGGCGTC 128

GLEAN_04439 Toll4 CCGTCCTTATAGTCCTGGCA TTTGTCCTTGTCGAGCTCCT 146

GLEAN_10851 IMD CCTCCAAGGGATGAAGTCAA TTGATCACACTGGCAAAAGC 137

GLEAN_00325 proPO1 ATTGTCGATAAAAATCGCCG GGGAAATAGGCTTCAGGGAG 149

GLEAN_14907 proPO2/3 TCCCCAACGACTACCTCAAC TCCAATTCGAGGATTTCACC 136

GLEAN_10349 lysozyme1 TAACCGATGACATTGCGTGT ACACAGCCCAGGCATTAAAC 84

GLEAN_10350 lysozyme2 CGCATTACAACACTGCTGCT CGTCCCGAATGTCGTTATCT 150

GLEAN_10351 lysozyme3 TCCTCTTCGTTTCGCTTTGT ACATCCATGTTGCCAGTTGA 123

GLEAN_10352 lysozyme4 TGCTATTTTCGCCTTTGCTT TCGGTGTTAAAAGCGGATTC 150

GLEAN_07738 attacin2 CGTAATTTTTGCACTCGCCT TGTAGTTTGAGGATTCCGGG 105

GLEAN_00500 cecropin3 GCTGTTCCCGTGGTTAAAAA ACTGGAGGCGCATACTGAAT 128

GLEAN_06250 defensin1 TAATTGCCCTTTTCTGCGTC ACAAGTGAAGCGTTTGACCC 80

GLEAN_10517 defensin2 TGTCGCTCTAGTTGCCCTTT GTTTAGCTTGACGGAGACGC 139

GLEAN_12469 defensin3 TGTCACACTAGTGATGGGGC ATCATTCTTTTGGTGTCCCG 117

defensin4 defensin4 CCATCCGAAAAGCGAAATTA ACATCATGCGTTTGGTATCG 83

GLEAN_05093 coleoptericin1 TTTGGCACTTTTTGCACTTG GGGATGTCCTGTTCTACGGA 120

GLEAN_08261 rpS3 TTGACGGTCTCATGATCCAC GTCCCACGGCAACATAATCT 122
